# Integrative Analysis of Disease Signatures Shows Inflammation Disrupts Juvenile Experience-Dependent Cortical Plasticity

**DOI:** 10.1523/ENEURO.0240-16.2016

**Published:** 2017-01-18

**Authors:** Milo R. Smith, Poromendro Burman, Masato Sadahiro, Brian A. Kidd, Joel T. Dudley, Hirofumi Morishita

**Affiliations:** 1 Department of Neuroscience, Icahn School of Medicine at Mount Sinai, New York, New York 10029; 2 Department of Genetics and Genomic Sciences, Icahn School of Medicine at Mount Sinai, New York, New York 10029; 3 Department of Psychiatry, Icahn School of Medicine at Mount Sinai, New York, New York 10029; 4 Department of Ophthalmology, Icahn School of Medicine at Mount Sinai, New York, New York 10029; 5 Mindich Child Health and Development Institute, Icahn School of Medicine at Mount Sinai, New York, New York 10029; 6 Graduate School of Biomedical Sciences, Icahn School of Medicine at Mount Sinai, New York, New York 10029; 7 Icahn Institute for Genomics and Multiscale Biology, Icahn School of Medicine at Mount Sinai, New York, New York 10029; 8 Friedman Brain Institute, Icahn School of Medicine at Mount Sinai, New York, New York 10029

**Keywords:** bioinformatics, critical period, inflammation, plasticity, transcriptome, visual cortex

## Abstract

Throughout childhood and adolescence, periods of heightened neuroplasticity are critical for the development of healthy brain function and behavior. Given the high prevalence of neurodevelopmental disorders, such as autism, identifying disruptors of developmental plasticity represents an essential step for developing strategies for prevention and intervention. Applying a novel computational approach that systematically assessed connections between 436 transcriptional signatures of disease and multiple signatures of neuroplasticity, we identified inflammation as a common pathological process central to a diverse set of diseases predicted to dysregulate plasticity signatures. We tested the hypothesis that inflammation disrupts developmental cortical plasticity *in vivo* using the mouse ocular dominance model of experience-dependent plasticity in primary visual cortex. We found that the administration of systemic lipopolysaccharide suppressed plasticity during juvenile critical period with accompanying transcriptional changes in a particular set of molecular regulators within primary visual cortex. These findings suggest that inflammation may have unrecognized adverse consequences on the postnatal developmental trajectory and indicate that treating inflammation may reduce the burden of neurodevelopmental disorders.

## Visual Abstract

**Figure:**
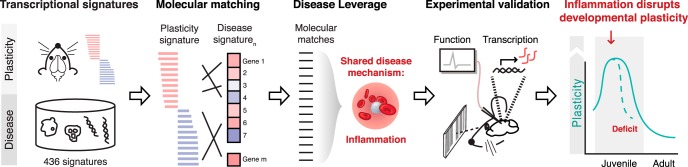

## Significance Statement

During childhood and adolescence, heightened neuroplasticity allows the brain to reorganize and adapt to its environment. Disruptions in these malleable phases can result in permanent neurodevelopmental disorders. To identify pathological mechanisms that disrupt developmental neuroplasticity, we applied a systematic computational screen of hundreds of diseases for their impact on neuroplasticity. We discovered that inflammation would putatively disrupt neuroplasticity and validated this hypothesis in an *in vivo* experimental mouse model of developmental cortical plasticity. This work suggests that inflammation during the childhood period could have unrecognized negative consequences on the neurodevelopmental trajectory.

## Introduction

During childhood and adolescence, the human brain undergoes tremendous reorganization during windows of heightened neuroplasticity. These windows of plasticity are critical periods that allow brain circuits to be refined by sensory and social experiences, which help to establish normal perception and higher cognitive function (Johnson and Newport, 1989; Nikolopoulos et al., 1999; Lewis and Maurer, 2005; Schorr et al., 2005; Nelson et al., 2007; Fox et al., 2010). Disruption of these critical periods can alter neural circuits that shape function and behavior, which may, in turn, contribute to a wide range of psychiatric and neurodevelopmental disorders, such as autism (Weinberger, 1987; LeBlanc and Fagiolini, 2011; Takesian and Hensch, 2013; Lee et al., 2014). Previous studies focused on several genes relevant to autism spectrum disorders (*MeCP2*, *Ube3a*, *Fmr1*) and identified marked disruptions in developmental cortical plasticity (Tropea et al., 2009; Yashiro et al., 2009; Harlow et al., 2010). To our knowledge, no studies have conducted a systematic evaluation of pathological mechanisms that may disrupt developmental plasticity. The goal of this study was to leverage the growing repository of publically available transcriptome data from diverse disease states to identify pathological processes with the capacity to disrupt developmental cortical plasticity.

To identify pathological processes that disrupt developmental plasticity, we designed an integrative bioinformatics approach that identifies disruptive pathways through systematic evaluation of molecular profiles of disease states in humans and animals. Our approach adapts molecular matching algorithms from computational drug repurposing (for review, see Hodos et al., 2016) to match transcriptional signatures of disease to those of neuroplasticity. To model plasticity, we leveraged the paradigmatic ocular dominance model of *in vivo* developmental plasticity (Wiesel and Hubel, 1963) and generated transcriptional signatures from primary visual cortex (V1). We matched plasticity and disease signatures to produce a diverse list of diseases ranked by their likelihood to dysregulate developmental plasticity. Across this ranked list, we sought to identify shared pathophysiology, rather than generate hypotheses about individual disease matches. To quantify shared pathophysiology, we developed and applied a novel Disease Leverage Analysis (DLA) that identifies shared molecular patterns of disease signatures to reveal novel disruptors of developmental plasticity. By examining shared pathophysiology, DLA identified a strong relationship between the molecular signatures of inflammation and developmental plasticity. We tested the hypothesis that inflammation disrupts developmental plasticity in the ocular dominance model of developmental V1 plasticity and found that functional, experience-dependent plasticity *in vivo* was suppressed by systemic inflammation. Our study demonstrates the utility of an integrative bioinformatics approach for identifying disruptors of developmental neuroplasticity and suggests that inflammation may be an unrecognized risk factor for neurodevelopmental disorders.

## Materials and Methods

### Animals

Male C57BL/6 mice (Charles River Laboratories) and *Lynx1*^−/−^ mice (Miwa et al., 2006; a gift from Dr. Nathaniel Heintz, Rockefeller University, New York, NY) were group housed (three to five animals per cage) under a standard 12 h light/dark cycle (lights on at 7:00 A.M., lights off at 7:00 P.M.) with constant temperature (23°C) and *ad libitum* access to food and water. The Institutional Animal Care and Use Committee at the Icahn School of Medicine at Mount Sinai approved all procedures involving animals.

### Substances

Lyophilized *Escherichia coli* lipopolysaccharide (LPS; 600,000 endotoxin units/mg; serotype 0127:B8, catalog #L5024, lot 073M4024V, Sigma-Aldrich) was reconstituted in sterile saline (0.9% NaCl) to yield a stock solution of 2 mg/ml, which was diluted with saline on the day of injection to yield a working concentration of 0.03 mg/ml.

### Plasticity signature generation

Transcriptomes were profiled with microarray to generate plasticity signatures. Experiment-naive juvenile C57BL/6 mice at postnatal day 29 (P29), adult *Lynx1*^−/−^ mice (>P60), and adult C57BL/6 mice (>P60; *n* = 3 each group) were anesthetized with isoflurane and cervically dislocated; bilateral V1 was removed, immediately frozen on dry ice, and stored at −80°C until processed. Total RNA was extracted from V1 using RNeasy Lipid Tissue Mini kit (Qiagen) and stored at −80°C. A total of 4.5 μg of cRNA was hybridized to Illumina WG-6 2.0 microarrays (750 ng/subarray). A juvenile plasticity signature was generated via differential expression analyses of juvenile versus adult V1 transcriptomes by first quantile normalizing probe-level data with limma (Smyth, 2005) and then computing rank-based differential expression with RankProd (Hong et al., 2006; both R packages available through the Bioconductor repository) to yield 193 unique mouse Entrez IDs. For downstream analysis, mouse Entrez IDs were mapped to human orthologs using the Mouse Genome Informatics homology reference to yield a 176 gene juvenile plasticity signature. We generated a *Lynx1*^−/−^ signature in an analogous manner to juvenile, to yield a *Lynx1*^−/−^ plasticity signature of 98 genes. Raw data for plasticity signatures is freely available at the Gene Expression Omnibus under accession number GSE89757.

### Molecular matching algorithm

To identify diseases that are predicted to dysregulate plasticity signature genes, we developed a molecular matching score, which is the sum of the absolute value of the rank-difference gene expression measure of disease signatures (for details of this expression measure, see Dudley et al., 2009) that intersect with neuroplasticity signature genes. The absolute value was chosen to simplify downstream interpretation. This score is similar in spirit to the approach taken by Zhang and Gant (2008), except in our implementation high scores indicate significant overlap between disease and plasticity signatures, whereas low scores indicate little or no overlap. To compare match scores (M) across diseases, we normalized the scores with *n* = 10,000 permutations of scores using $\frac{{\text{M}}_{actual}\;-\;{\overset{¯}{M}}_{perm}}{\sqrt{\frac{\sum_{i=1}^{n}{\left({\text{M}}_{perm_{i}}-{\overset{¯}{M}}_{perm}\right)}^{2}}{n-1}}}.$ *p* Values were estimated using the Generalized Pareto Distribution (Knijnenburg et al., 2009) on *n* permutations and were multiple test corrected using the method of Benjamini and Hochberg (1995).

### Disease Leverage Analysis

We developed DLA to infer pathological processes that are shared across diseases and predicted to dysregulate plasticity signature genes. For pathological processes, we used the 50 “hallmark” gene sets (MSigDb; Subramanian et al., 2005). We computed a pathology score for each hallmark gene set for each disease signature, for a total of 50 × 436 = 21,800 scores. A pathology score is the sum of the absolute value of the normalized disease signature gene expression that is shared with a hallmark gene set. The absolute value was chosen because the direction of effect for gene sets is not necessarily known. We next calculated a linear regression between the pathology scores for a specific gene set and the plasticity–disease molecular match scores. We estimated the *p* value for the association between the pathology scores and disease–plasticity scores (the β_1_ coefficient) by computing 20,000 permutations of pathology scores using gene sets the same length as the input gene set and then calculating the regression on the permuted scores. If $\overset{n}{\underset{i=1}{\sum }}[{𝟙}_{\left[\;|\;\text{β }1_{perm_{i}}|\;>\;|\;\text{β}1_{actual}\;|\;\right]}]\;>\;10$, where 𝟙 is the indicator function (i.e., the value is 1 when the conditional is satisfied and 0 otherwise) and *n* = the number of permutations, the *p* value was the empirical estimate: $\frac{\sum_{i=1}^{n}|\;\;\text{β}1_{perm_{i}}\;|\;>\;|\;\text{β}1_{actual}\;|}{n}$; otherwise, the Generalized Pareto Distribution was used to estimate the *p* value (Knijnenburg et al., 2009). Bonferroni’s method was used to correct for multiple hypothesis tests (denoted *p*_corrected_ in the text). To account for the probability of a large coefficient by chance, actual β_1_ coefficients were normalized by the permutated distribution of β_1_ according to $\frac{\text{β}1_{actual}\;-\;{\overset{¯}{\text{β1}}}_{perm}}{\sqrt{\frac{\sum_{i=1}^{n}{\left(\text{β}1_{perm_{i}}-{\overset{¯}{\text{β1}}}_{perm}\right)}^{2}}{n-1}}}.$ Positive β values are pathological processes associated with diseases that were predicted to disrupt plasticity signatures. Negative β values are pathological processes associated with diseases that are predicted to not disrupt plasticity signatures. To calculate enrichments of top DLA gene sets, we chose a conservative cutoff of *p*_corrected_ < 5 × 10^−5^ and then calculated the over-representation of inflammation gene sets among positive DLA associations. To do so, we used the hypergea (Bönn, 2016) R package, which uses a conditional maximum likelihood estimate to compute the odds ratio (OR) on adjusted cell counts (to avoid empty cells) and obtains two-sided *p* values from the hypergeometric distribution.

### Quantitative PCR

Experiment-naive juvenile mice (P26; *n* = 5/group) were lightly anesthetized with isoflurane to avoid additional stressors and injected intraperitoneally before 12:00 noon eastern standard time with a dose of LPS that does not cross the blood–brain barrier (Banks and Robinson, 2010; 300 μg/kg, ∼4.5 μg/mouse) or vehicle (150 μl of saline). Four hours later, mice were deeply anesthetized with isoflurane and decapitated, and bilateral V1 was removed under RNAse-free conditions, briefly rinsed in sterile saline (0.9% NaCl), immediately frozen on dry ice, and transferred to −80°C storage until processed. Total RNA was extracted from V1 using the RNeasy Lipid Tissue Mini Kit (Qiagen) and stored at −80°C. RNA yields ranged from 4.5 to 10 μg/sample and RNA integrity numbers ranged from 8.7 to 10 (mean, 9.8; SD, 0.32). Total V1 RNA was converted to cDNA using a High-Capacity cDNA Reverse Transcription Kit (Life Technologies). Quantitative PCR (qPCR) was performed by the Mount Sinai Quantitative PCR core facility using TaqMan probes (catalog numbers: *NogoR*: 00445861, *Lynx1*: 01204957_g1, *S100a8*: 00496696_g1, *Lrg1*: 01278767_m1, *Lcn2*: 01324470_m1, *PirB*: 01700366_m1, *Cldn5*: 00727012_s1, *Egr2*: 00456650_m1, *Npas4*: 01227866_g1, Il1: 00434228_m1, *Agmat*: 01348862_m1, *Ch25h*: 00515486_s1, *Alox12b*: 01325300_gH, *Evpl*: 01700609_m1, *Slc40a1*: 00489835_g1, *Arc*: 01204954_g1, *S100a9*: 00656925_m1, *H2D1/H2K1*: 04208017_mH, *BDNF*: 04230607_s1, *Nptx2*: 00479438_m1, and *Ppp3ca*: 01317678_m1, Applied Biosystems). Quantification of the fold change was derived via the –ΔΔ CT method (equivalent to a log2 fold change) and significance was computed with parametric *t* tests of the Δ CTs, given the approximately normal distribution of Δ CTs. To prioritize qPCR validations, we identified the most differentially dysregulated juvenile neuroplasticity genes by an independent LPS brain study (GSE3253) by subsetting with the LPS brain expression whose absolute expression statistic was ≥2 after conversion to *z*-score.

### *In vivo* electrophysiology

Under light isoflurane anesthesia, the contralateral eye of experiment-naive P26 mice was sutured, and the animal was immediately injected intraperitoneally with LPS (300 μg/kg, ∼4.5 μg/mouse) or vehicle (150 μl saline). Three days later, single-unit electrophysiological recordings were taken in binocular zone of V1 in response to visual stimuli presented to each eye separately (Gordon and Stryker, 1996). Briefly, recording was conducted under nembutal/chlorprothixene anesthesia. Visually evoked single-unit responses were recorded with 16-channel silicone probes (NeuroNexus) in response to a high-contrast single bar generated by visage system (Cambridge Research Systems). The signal was amplified and thresholded (OmniPlex, Plexon). To ensure single-unit isolation, the waveforms of recorded units were further examined off-line (Offline Sorter, Plexon). For each animal, approximately 3–10 single units were recorded in each of the four to six vertical penetrations spaced evenly (250 μm intervals) across the mediolateral extent of V1 to map the monocular and binocular zones and to avoid sampling bias. Monocular zone was identified when three consecutive units solely registered contralateral responses within a single penetration [ocular dominance score (ODS) of 1, see below for definition of ODS]. Secondary visual cortex was identified by the reversal of retinotopy seen as the electrode was moved into the secondary visual cortex (Gordon and Stryker, 1996). Mice that experienced opening of the sutured eye or had poor recordings (<10 cells/mouse or <3 penetrations/mouse or lack of positive identification of monocular zone and secondary visual cortex) were excluded from further study. To analyze the electrophysiology data, normalized ocular dominance index (ODI) of single neurons was computed by a custom-made MATLAB code by peristimulus time histogram analysis of peak to baseline spiking activity in response to each eye: {[Peak(ipsi) − baseline(ipsi)] − [Peak(contra) − baseline(contra)]}/{[Peak(ipsi) − baseline(ipsi)] + [Peak(contra) − baseline(contra)]}, which produces a range of [−1,+1] where −1 is a completely contralateral dominated cell and +1 is a completely ipsilateral dominated cell. ODI is linearly transformed by assigning [−1.0, −0.5) = **1**, [−0.5, −0.3) = **2**, [−0.3, −0.1) = **3**, [−0.1, +0.1] = **4**, (+0.1, +0.3] = **5**, (+0.3, +0.5] = **6**, (+0.5, +1.0] = **7** to produce the ODS. Finally, the contralateral bias index (CBI), a monocular weighted, animal-level summary statistic, is computed from the ODS, as follows: [(*n***1** − *n***7**) + 2/3(*n***2** − *n***6**) + 1/3(*n***3** − *n***5**) + *N*]/2*N*, where *N* = total number of cells and *n***x** = number of cells corresponding to ODS of **x**. Thus, a CBI value of 0.7 is contralateral dominant, and a CBI value of 0.4 is ipsilateral dominant. For statistical comparison of ocular dominance, ODSs of single neurons were plotted as a proportion histogram and compared via a nonparametric χ^2^ test, and CBI values of single animals were compared via a *t* test. Saline-treated juvenile animals (age P26) were the comparison group. The experimenter was blind to the sample group. Sample sizes were statistically estimated prior to undertaking experimental work to be *n* = 6 per group, assuming the effect sizes seen in previous relevant work.

### Statistical analysis

All statistical and computational analyses conducted with R (version 3.2.2) and Python (version 2.7.10). Parametric Welch *t* tests were two sided, unless otherwise noted. Sample sizes (denoted *n*) always indicate the number of mice. The influenza 95% confidence interval (CI) for the incidence rate ratio was estimated using the Katz log approach (Fagerland et al., 2015) $\left(e^{log(IRR)\pm \sqrt{\frac{1}{a}+\frac{1}{b}-\frac{1}{m}-\frac{1}{n}}}\right)$, where *a* and *b* are the successes and *m* and *n* are the totals (totals determined by dividing the successes by the published incidence rates. The detail of statistical analysis is shown in Table 1.

**Table 1:** Statistics

Subscript	Data structure	Type of test	Power
a	Hypergeometric	Fisher's exact	95% CI = 1.4–491.2
b	Hypergeometric	Fisher's exact	95% CI = 23.8–58.0
c	Hypergeometric	Fisher's exact	95% CI = 3.2–1229.8
d	Approximately normal	Welch *t* test	95% CI = −3.3 to −1.6, alternative hypothesis: true difference in means is not equal to 0
e	Non-normal	Empirical *p* value	NA
f	Non-normal	Spearman rank correlation	NA
g	Approximately normal	Welch *t* test	NA; no specific *p* values reported, rather a group of genes under a specified *p* value threshold
h	Approximately normal	Welch *t* test	95% CI = −3.0 to −0.1, alternative hypothesis: true difference in means is not equal to 0
i	Approximately normal	Welch *t* test	95% CI = −1.3 to −0.1, alternative hypothesis: true difference in means is not equal to 0
j	Approximately normal	Welch *t* test	95% CI = 0.3–1.6, alternative hypothesis: true difference in means is not equal to 0
k	Approximately normal	Welch *t* test	NA; no specific *p* values reported, rather a group of genes above a specified *p* value threshold (e.g., not significant)
l	Approximately normal	Welch *t* test, one-sided (due to specific prior hypothesis as to direction of effect; results still significant using a two-sided test at a threshold of α = 0.05)	95% CI = 0.1 to ∞], alternative hypothesis: true difference in means is not equal to 0
m	Non-normal	χ^2^ test of ODS counts	NA

## Results

### Molecular matching between plasticity and disease signatures

To enable molecular matching between plasticity and disease, we compared V1 transcriptomes of juvenile wild-type mice, during the critical period of elevated ocular dominance plasticity in V1 (Gordon and Stryker, 1996), with adult wild-type mice with reduced plasticity, to identify a differential expression signature of 176 genes (Fig. 1a; Table 2). We computationally matched this signature to 436 disease signatures derived from public microarray data using a previously described method (Dudley et al., 2009; Fig. 1a). This systematic method applies a rank-based molecular matching algorithm to determine the molecular concordance between the plasticity signature and a given disease signature, where high scores indicate plasticity genes are significantly dysregulated by the disease and low scores indicate that the disease has no impact on plasticity genes (for details, see Materials and Methods; Fig. 1b). The molecular matching procedure produced a list of 436 diseases ordered by their prediction to disrupt the plasticity signature. Interestingly, highly ranked diseases included not only brain disorders known to disrupt plasticity, such as Huntington's disease (Usdin et al., 1999; Murphy et al., 2000; Milnerwood et al., 2006), but also non-neurologic disorders (e.g., bacterial infections, inflammatory bowel disease, metabolic diseases), suggesting that a broad range of disease states may impact molecular pathways involved in plasticity.

**Figure 1.**
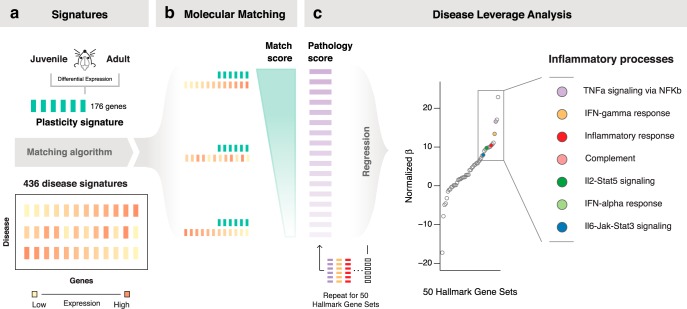
Diseases that dysregulate the juvenile plasticity signature are associated with inflammatory processes. ***a***, ***b***, Juvenile plasticity signature of 176 genes (represented by green bars) generated by differential expression of P29 vs >P60 C57BL/6 male mice primary visual cortex was computationally matched to 436 disease signatures (represented by orange bars; ***a***) using rank-based molecular matching where large scores indicate shared transcriptional phenotype (***b***). ***c***, DLA systematically identified processes associated to diseases that dysregulate the plasticity signature. Seven of the 14 largest associations were inflammation-related gene sets, and 7 of 7 of inflammation-related gene sets were strongly associated with plasticity (all at *p*_corrected_ < 5 × 10^−5^: OR = 25.8, 95% CI = 1.4–491.2, *p* = 2.7 × 10^−3^, Fisher’s exact test). See Figure 1-1 for source DLA data.

**Table 2:** Juvenile plasticity signature

RP	FC	pfp	*p* value	Probe_id	Symbol	mm_Entrez_ID	hs_Entrez_ID	Gene_name
2.8187	3.7313	0	0	ILMN_2641456	Pcp2	18545	126006	Purkinje cell protein 2 (L7)
3.2916	3.422	0	0	ILMN_2503052	Tnnc1	21924	7134	Troponin C, cardiac/slow skeletal
4.2819	3.1519	0	0	ILMN_2794645	Cyr61	16007	3491	Cysteine-rich protein 61
13.632	2.5389	0	0	ILMN_1251414	Npas4	225872	266743	Neuronal PAS domain protein 4
28.3181	2.375	0	0	ILMN_2597827	Arc	11838	23237	Activity-regulated cytoskeletal-associated protein
15.7472	2.3415	0	0	ILMN_1230397	A630064P09Rik	NA	NA	NA
15.7373	2.331	0	0	ILMN_2622983	Dusp1	19252	1843	Dual-specificity phosphatase 1
14.4292	2.3046	0	0	ILMN_3160970	Gpr17	574402	2840	G-protein-coupled receptor 17
16.9658	2.2123	0	0	ILMN_1250438	Marcksl1	17357	65108	MARCKS-like 1
21.67	2.1606	0	0	ILMN_2710253	Cyr61	16007	3491	Cysteine-rich protein 61
20.9478	2.1439	0	0	ILMN_1217458	8430403J19Rik	NA	NA	NA
23.8738	2.111	0	0	ILMN_1220034	Junb	16477	3726	jun B proto-oncogene
27.1365	2.0864	0	0	ILMN_2744890	Gadd45g	23882	10912	Growth arrest and DNA-damage-inducible 45 gamma
25.9586	2.0549	0	0	ILMN_1227299	Mbp	17196	4155	Myelin basic protein
29.3188	2.0001	0	0	ILMN_1239557	Ugt8a	22239	7368	UDP galactosyltransferase 8A
27.0514	1.9972	0	0	ILMN_2619767	Pdlim2	213019	64236	PDZ and LIM domain 2
32.1696	1.9948	0	0	ILMN_2707616	Col22a1	69700	169044	Collagen, type XXII, alpha 1
32.3997	1.9656	0	0	ILMN_1221178	Pdlim2	213019	64236	PDZ and LIM domain 2
38.5983	1.9267	5.00E-04	0	ILMN_2463181	Tnc	21923	3371	Tenascin C
37.2206	1.9239	5.00E-04	0	ILMN_2810882	Ppic	19038	5480	Peptidylprolyl isomerase C
41.0256	1.9217	5.00E-04	0	ILMN_2615034	Mog	17441	4340	Myelin oligodendrocyte glycoprotein
37.2906	1.9117	5.00E-04	0	ILMN_2653205	Gp1bb	14724	2812	Glycoprotein Ib, beta polypeptide
43.0584	1.9067	4.00E-04	0	ILMN_1212702	Hba-a1	NA	NA	NA
47.3908	1.8855	8.00E-04	0	ILMN_1240973	Slc29a4	243328	222962	Solute carrier family 29 (nucleoside transporters), member 4
145.4493	1.8339	0.0108	0	ILMN_1253365	Lypd1	71111	2863	G-protein-coupled receptor 39
49.1546	1.832	8.00E-04	0	ILMN_2802263	Cnp	12799	1267	2',3'-cyclic nucleotide 3' phosphodiesterase
89.4321	1.8212	0.0037	0	ILMN_2491182	A130010C12Rik	NA	NA	NA
85.0435	1.8206	0.0024	0	ILMN_2766894	Enpp6	320981	133121	Ectonucleotide pyrophosphatase/phosphodiesterase 6
60.0087	1.7971	8.00E-04	0	ILMN_1223244	Hbb-b1	NA	NA	NA
71.2786	1.7908	0.0012	0	ILMN_2906855	Ky	16716	339855	Kyphoscoliosis peptidase
63.327	1.7828	7.00E-04	0	ILMN_2880906	Pdlim2	213019	64236	PDZ and LIM domain 2
79.6764	1.7799	0.0024	0	ILMN_1256343	H19	14955	NA	H19, imprinted maternally expressed transcript
100.4221	1.7744	0.0048	0	ILMN_1252953	Cbln1	12404	869	Cerebellin 1 precursor protein
62.1078	1.7686	7.00E-04	0	ILMN_2617162	Mlp	17357	65108	MARCKS-like 1
83.6683	1.7607	0.0025	0	ILMN_2955919	Mcam	84004	4162	Melanoma cell adhesion molecule
70.6284	1.7566	0.001	0	ILMN_1259536	Mog	17441	4340	Myelin oligodendrocyte glycoprotein
68.3987	1.753	0.001	0	ILMN_2754447	Mkrn3	22652	7681	Makorin, ring finger protein, 3
68.3151	1.7464	0.001	0	ILMN_2597606	Gjc2	118454	57165	Gap junction protein, gamma 2
95.7042	1.7345	0.0044	0	ILMN_2544056	Hbb-b1	100503605	3043	Hemoglobin, beta adult s chain
76.7826	1.7239	0.0024	0	ILMN_1242456	Kank1	107351	23189	KN motif and ankyrin repeat domains 1
82.1412	1.7197	0.0026	0	ILMN_1237021	Mag	17136	4099	Myelin-associated glycoprotein
107.5658	1.7077	0.0049	0	ILMN_2675874	Alas2	11656	212	Aminolevulinic acid synthase 2, erythroid
105.2858	1.6961	0.0049	0	ILMN_3161282	Dpysl5	65254	56896	Dihydropyrimidinase-like 5
104.9667	1.6801	0.005	0	ILMN_1216452	Hbb-b1	NA	NA	NA
101.7441	1.677	0.0048	0	ILMN_2735184	Col18a1	12822	80781	Collagen, type XVIII, alpha 1
165.7567	1.6719	0.0137	1.00E-04	ILMN_1241293	Cldn5	12741	7122	Claudin 5
104.234	1.67	0.0051	0	ILMN_2991389	Ly6g6e	70274	NA	Lymphocyte antigen 6 complex, locus G6E
109.9056	1.6629	0.006	0	ILMN_3105563	Dmkn	73712	93099	Dermokine
100.5225	1.6611	0.0046	0	ILMN_1259039	Sox8	NA	NA	NA
119.8152	1.6562	0.0082	0	ILMN_1236718	Hbb-b1	NA	NA	NA
107.4491	1.6543	0.005	0	ILMN_1234698	Tspan2	70747	10100	Tetraspanin 2
114.2191	1.651	0.0071	0	ILMN_2457585	Trp53inp2	68728	58476	Transformation-related protein 53-inducible nuclear protein 2
127.672	1.6497	0.0091	0	ILMN_2977558	Dapk2	13143	23604	Death-associated protein kinase 2
115.4908	1.6484	0.0078	0	ILMN_2777359	Serpinh1	12406	871	Serine (or cysteine) peptidase inhibitor, clade H, member 1
133.2106	1.6468	0.009	0	ILMN_2757125	Prc1	233406	9055	Protein regulator of cytokinesis 1
168.1391	1.645	0.0138	1.00E-04	ILMN_2440194	5330423I11Rik	NA	NA	NA
133.3424	1.6326	0.0088	0	ILMN_2903945	Gadd45g	23882	10912	Growth arrest and DNA-damage-inducible 45 gamma
237.4537	1.6307	0.0286	2.00E-04	ILMN_3159435	Mid1	17318	NA	Midline 1
121.1858	1.6264	0.0083	0	ILMN_2598103	Emp2	13731	2013	Epithelial membrane protein 2
129.3051	1.6258	0.0091	0	ILMN_1215632	Marcksl1	17357	65108	MARCKS-like 1
132.4496	1.6257	0.0091	0	ILMN_3144289	Traf3	22031	7187	TNF receptor-associated factor 3
139.3631	1.6219	0.0092	0	ILMN_2675000	4930511J11Rik	74720	283953	Claudin 26
125.9	1.6217	0.0091	0	ILMN_2439638	Traf3	22031	7187	TNF receptor-associated factor 3
264.9656	1.6146	0.0379	2.00E-04	ILMN_2623983	Egr2	13654	1959	Early growth response 2
167.9258	1.6117	0.0141	1.00E-04	ILMN_2506428	Ky	16716	339855	Kyphoscoliosis peptidase
149.169	1.6067	0.0109	0	ILMN_2545963	Hbb-b1	NA	NA	NA
147.5637	1.6053	0.0108	0	ILMN_2769490	5430435G22Rik	226421	338382	RIKEN cDNA 5430435G22 gene
216.6505	1.6037	0.0234	1.00E-04	ILMN_2443330	Ttr	22139	7276	Transthyretin
147.9521	1.6023	0.0111	0	ILMN_2467151	Cyp11a1	NA	NA	NA
177.2045	1.6005	0.0155	1.00E-04	ILMN_1245549	6330404C01Rik	80982	57214	Cell migration inducing protein, hyaluronan binding
156.4386	1.5987	0.0116	0	ILMN_1235571	Cyr61	16007	3491	Cysteine-rich protein 61
151.0446	1.5966	0.0108	0	ILMN_2784078	Mmp15	17388	4324	Matrix metallopeptidase 15
128.5692	1.592	0.0091	0	ILMN_1234099	Fermt1	241639	55612	Fermitin family homolog 1 (*Drosophila*)
153.0722	1.591	0.0109	0	ILMN_2701891	Marcksl1	17357	65108	MARCKS-like 1
169.069	1.5888	0.0138	1.00E-04	ILMN_1255462	Hbb-b1	NA	NA	NA
238.1918	1.5774	0.0283	2.00E-04	ILMN_2750515	Fos	14281	2353	FBJ osteosarcoma oncogene
179.4286	1.5767	0.0157	1.00E-04	ILMN_1258028	Gal3st1	53897	9514	Galactose-3-O-sulfotransferase 1
168.0194	1.5754	0.0139	1.00E-04	ILMN_2621544	2700060E02Rik	18074	22795	Nidogen 2
179.1935	1.5706	0.0159	1.00E-04	ILMN_2711163	Ctsk	13038	1513	Cathepsin K
203.9522	1.5702	0.021	1.00E-04	ILMN_2965660	Apcdd1	494504	147495	Adenomatosis polyposis coli downregulated 1
186.0794	1.5661	0.0162	1.00E-04	ILMN_2778722	Ppapdc1a	381925	196051	Phosphatidic acid phosphatase type 2 domain containing 1A
183.6942	1.5565	0.0158	1.00E-04	ILMN_1246139	Cldn11	18417	5010	Claudin 11
183.6736	1.5551	0.0161	1.00E-04	ILMN_2838308	Fmo1	14261	2326	Flavin-containing monooxygenase 1
209.5265	1.5551	0.0219	1.00E-04	ILMN_2769777	Msc	17681	9242	Musculin
256.1212	1.5496	0.0346	2.00E-04	ILMN_2703138	Tmem125	230678	128218	Transmembrane protein 125
204.0502	1.5465	0.0207	1.00E-04	ILMN_2623184	Nkiras2	71966	28511	NF-κB inhibitor interacting Ras-like protein 2
202.9992	1.5454	0.0207	1.00E-04	ILMN_2650447	Col23a1	237759	91522	Collagen, type XXIII, alpha 1
215.8733	1.545	0.0231	1.00E-04	ILMN_1239117	Hbb-b1	NA	NA	NA
221.9056	1.5432	0.0251	1.00E-04	ILMN_2638473	1190003M12Rik	68888	NA	Gastrokine 3
202.5197	1.5425	0.0208	1.00E-04	ILMN_2753342	Hapln1	12950	1404	Hyaluronan and proteoglycan link protein 1
210.5965	1.5409	0.022	1.00E-04	ILMN_2759371	Fgfbp1	14181	9982	Fibroblast growth factor binding protein 1
235.9454	1.5331	0.0284	2.00E-04	ILMN_3097381	Mobp	17433	4336	Myelin-associated oligodendrocytic basic protein
217.9308	1.5305	0.0236	1.00E-04	ILMN_2909782	Rras2	66922	22800	Related RAS viral (r-ras) oncogene homolog 2
229.1399	1.5299	0.0263	1.00E-04	ILMN_2472451	Traf4	22032	9618	TNF receptor-associated factor 4
271.8732	1.5286	0.0398	2.00E-04	ILMN_3162060	EG574403	574403	100131897	Family with sequence similarity 196, member B
233.6047	1.5221	0.0277	2.00E-04	ILMN_2747923	Slc40a1	53945	30061	Solute carrier family 40 (iron-regulated transporter), member 1
227.3295	1.5175	0.0263	1.00E-04	ILMN_2517041	Uhrf1	18140	29128	Ubiquitin-like, containing PHD and RING finger domains, 1
280.3468	1.5086	0.0429	3.00E-04	ILMN_1241168	Dok4	114255	55715	Docking protein 4
254.0057	1.5079	0.0341	2.00E-04	ILMN_2625854	2310016C16Rik	69590	493869	Glutathione peroxidase 8 (putative)
256.2349	1.5077	0.0342	2.00E-04	ILMN_1229726	Fibcd1	98970	84929	Fibrinogen C domain containing 1
304.0763	1.5074	0.0497	3.00E-04	ILMN_2522884	9930105H17Rik	NA	NA	NA
270.2753	1.5045	0.0393	2.00E-04	ILMN_2815506	Gamt	14431	2593	Guanidinoacetate methyltransferase
262.3132	1.5016	0.0369	2.00E-04	ILMN_2778111	Etv4	18612	2118	ets variant 4
288.425	1.4979	0.0449	3.00E-04	ILMN_2604224	Sema5a	20356	9037	Sema domain, seven thrombospondin repeats (type 1 and type 1-like), transmembrane domain (TM) and short cytoplasmic domain, (semaphorin) 5A
268.7263	1.4966	0.039	2.00E-04	ILMN_2688236	Atp2a3	53313	489	ATPase, Ca++ transporting, ubiquitous
284.2455	1.4958	0.044	3.00E-04	ILMN_1236788	Igfbp2	16008	3485	Insulin-like growth factor-binding protein 2
287.3425	1.492	0.0451	3.00E-04	ILMN_2737479	Slc12a9	83704	56996	Solute carrier family 12 (potassium/chloride transporters), member 9
274.5651	1.4905	0.041	3.00E-04	ILMN_1257097	Cnp	12799	1267	2',3'-cyclic nucleotide 3' phosphodiesterase
288.7593	1.4897	0.0446	3.00E-04	ILMN_1219025	9030409G11Rik	71529	23254	Kazrin, periplakin interacting protein
297.6994	1.4891	0.0481	3.00E-04	ILMN_1251524	Them4	75778	117145	Thioesterase superfamily member 4
303.8564	1.4877	0.0501	3.00E-04	ILMN_1226329	Cd93	17064	22918	CD93 antigen
305.5889	1.4821	0.0492	3.00E-04	ILMN_3126277	Palmd	114301	54873	Palmdelphin
299.226	1.4801	0.0486	3.00E-04	ILMN_2655204	Apc	11789	324	Adenomatosis polyposis coli
303.6034	1.4788	0.0505	3.00E-04	ILMN_2726030	AB023957	NA	NA	NA
304.1851	1.476	0.0493	3.00E-04	ILMN_2772155	LOC100045780	11492	8728	A disintegrin and metallopeptidase domain 19 (meltrin beta)
326.2746	−1.5473	0.0492	4.00E-04	ILMN_2939681	Lyzs	17110	4069	Lysozyme 1
324.3234	−1.5513	0.0485	4.00E-04	ILMN_2718266	Fkbp5	14229	2289	FK506 binding protein 5
300.7726	−1.5755	0.0398	3.00E-04	ILMN_2775885	Calm2	12314	NA	Calmodulin 2
278.2959	−1.5868	0.0343	2.00E-04	ILMN_1224363	Slc12a5	57138	57468	Solute carrier family 12, member 5
281.5906	−1.6113	0.0347	3.00E-04	ILMN_1251998	Gm765	330390	NA	Predicted gene 765
226.9486	−1.6145	0.0249	1.00E-04	ILMN_2488510	Ppm1k	243382	152926	Protein phosphatase 1K (PP2C domain containing)
308.258	−1.6168	0.0431	3.00E-04	ILMN_1248368	Mat2a	232087	4144	Methionine adenosyltransferase II, alpha
275.8602	−1.6184	0.0343	2.00E-04	ILMN_2669088	4930461P20Rik	78244	134218	DNAJ (Hsp40) homolog, subfamily C, member 21
253.8942	−1.62	0.0298	2.00E-04	ILMN_2435835	Evpl	14027	2125	Envoplakin
197.6328	−1.6297	0.0211	1.00E-04	ILMN_2602387	Nr1d2	353187	9975	Nuclear receptor subfamily 1, group D, member 2
311.5841	−1.6329	0.0428	3.00E-04	ILMN_2734000	Elavl4	15572	1996	ELAV (embryonic lethal, abnormal vision, *Drosophila*)-like 4 (Hu antigen D)
311.1495	−1.6404	0.043	3.00E-04	ILMN_2859032	Gfod1	328232	54438	Glucose-fructose oxidoreductase domain containing 1
253.0893	−1.6455	0.0299	2.00E-04	ILMN_2445958	Tssc8	63830	NA	KCNQ1 overlapping transcript 1
258.1943	−1.6504	0.0299	2.00E-04	ILMN_2419660	mtDNA_ND4L	NA	NA	NA
215.9199	−1.6523	0.0237	1.00E-04	ILMN_1221817	Cd74	16149	972	CD74 antigen (invariant polypeptide of MHC, class II antigen-associated)
308.2304	−1.6595	0.0434	3.00E-04	ILMN_2545149	Nos1ap	70729	NA	Nitric oxide synthase 1 (neuronal) adaptor protein
256.5251	−1.6631	0.0294	2.00E-04	ILMN_1219573	C130072A16Rik	105727	81539	Solute carrier family 38, member 1
292.3656	−1.6664	0.0367	3.00E-04	ILMN_2475376	BC044804	NA	NA	NA
229.2861	−1.6683	0.0251	1.00E-04	ILMN_1253544	2900060B14Rik	68204	NA	RIKEN cDNA 2900060B14 gene
318.4001	−1.6694	0.0454	4.00E-04	ILMN_1246494	LOC381445	26422	26960	Neurobeachin
282.6027	−1.6717	0.0345	3.00E-04	ILMN_2666980	BDNF	12064	627	BDNF
219.961	−1.6722	0.0236	1.00E-04	ILMN_1231445	Inmt	21743	11185	Indolethylamine N-methyltransferase
240.372	−1.6762	0.0269	2.00E-04	ILMN_1240202	Fnip1	216742	96459	Folliculin-interacting protein 1
269.2034	−1.6764	0.033	2.00E-04	ILMN_2987863	Per2	18627	8864	Period circadian clock 2
201.3209	−1.677	0.0212	1.00E-04	ILMN_3157692	Ankrd35	213121	148741	Ankyrin repeat domain 35
310.3109	−1.679	0.0432	3.00E-04	ILMN_1218471	3-Sep	24050	55964	Septin 3
229.1441	−1.6798	0.0254	1.00E-04	ILMN_1229216	Zbtb16	235320	7704	Zinc finger and BTB domain containing 16
264.7303	−1.6841	0.0316	2.00E-04	ILMN_1253985	A330021D07Rik	NA	NA	NA
171.7223	−1.6861	0.0163	1.00E-04	ILMN_1235966	Alox12b	11686	242	Arachidonate 12-lipoxygenase, 12R type
253.9144	−1.6861	0.0295	2.00E-04	ILMN_1245687	Ash1l	192195	55870	ash1 (absent, small, or homeotic)-like (Drosophila)
294.7856	−1.6875	0.0375	3.00E-04	ILMN_2674890	Tbl1x	21372	6907	Transducin (beta)-like 1 X-linked
267.8862	−1.6889	0.0328	2.00E-04	ILMN_2828916	Frmd6	319710	122786	FERM domain containing 6
254.4102	−1.6932	0.0293	2.00E-04	ILMN_1228077	6330437B22Rik	78283	256714	MAP7 domain containing 2
282.4957	−1.6935	0.0347	3.00E-04	ILMN_2589525	Cpeb3	208922	22849	Cytoplasmic polyadenylation element binding protein 3
277.0563	−1.6955	0.0345	2.00E-04	ILMN_2466121	Twistnb	28071	221830	TWIST neighbor
250.5161	−1.6978	0.029	2.00E-04	ILMN_2514631	scl0002315.1_12	217869	1983	Eukaryotic translation initiation factor 5
186.3783	−1.7007	0.0195	1.00E-04	ILMN_2493030	2310043N10Rik	66961	NA	Nuclear paraspeckle assembly transcript 1 (non-protein coding)
263.9285	−1.7033	0.0315	2.00E-04	ILMN_2677270	Peg3	18616	5178	Paternally expressed 3
239.6678	−1.7039	0.0271	2.00E-04	ILMN_1214405	Cnksr2	245684	22866	Connector enhancer of kinase suppressor of Ras 2
253.7716	−1.7042	0.03	2.00E-04	ILMN_2763404	Nrxn3	18191	NA	Neurexin III
182.2435	−1.7079	0.0186	1.00E-04	ILMN_1246861	Ctss	13040	1520	Cathepsin S
278.7674	−1.7082	0.0341	2.00E-04	ILMN_1239608	Arid4a	238247	5926	AT-rich interactive domain 4A (RBP1-like)
242.1379	−1.7088	0.0269	2.00E-04	ILMN_2745614	Fam134b	66270	54463	Family with sequence similarity 134, member B
273.3035	−1.7094	0.0336	2.00E-04	ILMN_2762701	Scn1a	20265	6323	Sodium channel, voltage-gated, type I, alpha
235.4555	−1.7097	0.0263	2.00E-04	ILMN_1243910	Zfp292	30046	23036	Zinc finger protein 292
182.6041	−1.71	0.0184	1.00E-04	ILMN_2690603	Spp1	20750	6696	Secreted phosphoprotein 1
262.6651	−1.7167	0.0313	2.00E-04	ILMN_2733314	Rgs7bp	52882	401190	Regulator of G-protein signaling 7 binding protein
218.5161	−1.7173	0.0237	1.00E-04	ILMN_2593368	Mat2a	232087	4144	Methionine adenosyltransferase II, alpha
248.3251	−1.7218	0.0284	2.00E-04	ILMN_2525034	Cc1	12421	9821	RB1-inducible coiled-coil 1
244.6249	−1.7232	0.0273	2.00E-04	ILMN_2713008	C030011O14Rik	215708	374986	Family with sequence similarity 73, member A
244.4499	−1.7244	0.0276	2.00E-04	ILMN_1228020	1500010G04Rik	NA	NA	NA
214.8649	−1.7247	0.0242	1.00E-04	ILMN_2664706	Chic1	12212	53344	Cysteine-rich hydrophobic domain 1
224.3588	−1.7256	0.0252	1.00E-04	ILMN_1251488	A430041B07Rik	328108	23116	Family with sequence similarity 179, member B
167.7266	−1.7283	0.0158	1.00E-04	ILMN_2651054	LOC100047173	269589	84958	Synaptotagmin-like 1
233.1724	−1.7313	0.0262	2.00E-04	ILMN_2713004	C030011O14Rik	215708	374986	Family with sequence similarity 73, member A
224.4289	−1.7346	0.0249	1.00E-04	ILMN_1233554	Pbrm1	66923	55193	Polybromo 1
215.0498	−1.7355	0.0239	1.00E-04	ILMN_2669461	Bbx	70508	56987	Bobby sox homolog (*Drosophila*)
220.6021	−1.7382	0.0238	1.00E-04	ILMN_1218712	Jph4	NA	NA	NA
229.662	−1.7394	0.0248	1.00E-04	ILMN_1231596	Mtap7	NA	NA	NA
217.0292	−1.7397	0.0237	1.00E-04	ILMN_2492395	2900064A13Rik	NA	NA	NA
194.6882	−1.7416	0.0213	1.00E-04	ILMN_1218051	Iqgap2	544963	10788	IQ motif containing GTPase activating protein 2
224.9623	−1.7422	0.0249	1.00E-04	ILMN_1220626	2010007K12Rik	NA	NA	NA
193.5535	−1.7437	0.0213	1.00E-04	ILMN_2689307	Spnb2	20742	6711	Spectrin β, non-erythrocytic 1
211.5491	−1.7446	0.0235	1.00E-04	ILMN_2594593	Mpp5	56217	64398	Membrane protein, palmitoylated 5 (MAGUK p55 subfamily member 5)
196.6783	−1.7461	0.0214	1.00E-04	ILMN_2541675	LOC382128	319675	85459	RIKEN cDNA 5830418K08 gene
225.9716	−1.7473	0.0251	1.00E-04	ILMN_1230605	Gm336	212285	116984	ArfGAP with RhoGAP domain, ankyrin repeat and PH domain 2
172.8921	−1.7587	0.0162	1.00E-04	ILMN_2803674	S100a9	20202	6280	S100 calcium binding protein A9 (calgranulin B)
192.6629	−1.759	0.0213	1.00E-04	ILMN_3072536	Eif5	217869	1983	Eukaryotic translation initiation factor 5
138.7531	−1.7606	0.0112	0	ILMN_1255416	Ly6a	110454	NA	Lymphocyte antigen 6 complex, locus A
206.7346	−1.7624	0.0217	1.00E-04	ILMN_2596077	2810474O19Rik	67246	55196	RIKEN cDNA 2810474O19 gene
190.8274	−1.7627	0.0211	1.00E-04	ILMN_1237335	A730028C12Rik	NA	NA	NA
197.1239	−1.7662	0.0214	1.00E-04	ILMN_2593230	Mllt4	17356	4301	Myeloid/lymphoid or mixed-lineage leukemia (trithorax homolog, Drosophila); translocated to, 4
200.8213	−1.769	0.0212	1.00E-04	ILMN_2595282	2600005C20Rik	72462	23076	Ribosomal RNA processing 1 homolog B (S. cerevisiae)
200.7106	−1.7702	0.0215	1.00E-04	ILMN_2481389	Zfp326	54367	284695	Zinc finger protein 326
164.0235	−1.774	0.0155	1.00E-04	ILMN_1220828	2900075B16Rik	78506	286097	Mitochondrial calcium uptake family, member 3
155.2155	−1.7756	0.0142	0	ILMN_2571934	D030034I04Rik	105727	81539	Solute carrier family 38, member 1
176.004	−1.7781	0.0166	1.00E-04	ILMN_1229727	Gpr123	52389	84435	G-protein-coupled receptor 123
175.0744	−1.7835	0.0164	1.00E-04	ILMN_1237059	Kcna1	16485	3736	Potassium voltage-gated channel, shaker-related subfamily, member 1
152.5807	−1.7867	0.014	0	ILMN_2512204	mt-Nd4l	NA	NA	NA
191.3641	−1.7915	0.021	1.00E-04	ILMN_1236941	Csnk2a1-rs3	12995	1457	Casein kinase 2, alpha 1 polypeptide
117.9399	−1.796	0.0095	0	ILMN_2629112	Asah3l	230379	340485	Alkaline ceramidase 2
154.7781	−1.7976	0.0144	0	ILMN_2423249	2010321I05Rik	233833	27327	Trinucleotide repeat containing 6a
169.376	−1.7979	0.0157	1.00E-04	ILMN_1260446	Ttc3	22129	7267	Tetratricopeptide repeat domain 3
159.9385	−1.8005	0.0148	0	ILMN_3151492	Ankrd12	106585	23253	ankyrin repeat domain 12
159.7083	−1.8008	0.0149	0	ILMN_2747381	Ddx24	27225	57062	DEAD (Asp-Glu-Ala-Asp) box polypeptide 24
117.7708	−1.806	0.0094	0	ILMN_2746797	Hsf4	26386	3299	Heat shock transcription factor 4
154.9416	−1.8113	0.0141	0	ILMN_1227149	Meg3	17263	NA	Maternally expressed 3
154.4618	−1.8188	0.0141	0	ILMN_2544603	2610015J01Rik	67039	NA	RNA binding motif protein 25
149.6626	−1.8218	0.0137	0	ILMN_1234357	A230057E24Rik	NA	NA	NA
160.7228	−1.8235	0.0145	0	ILMN_2737867	Mtap1b	17755	4131	Microtubule-associated protein 1B
146.4398	−1.8285	0.0132	0	ILMN_2657911	Cnot4	53621	4850	CCR4-NOT transcription complex, subunit 4
137.4716	−1.8312	0.0115	0	ILMN_2654932	Pdap1	231887	11333	PDGFA-associated protein 1
149.0718	−1.8342	0.0133	0	ILMN_2542231	LOC382157	228005	9360	Peptidyl-prolyl isomerase G (cyclophilin G)
136.8161	−1.8372	0.0118	0	ILMN_1256701	2900016B01Rik	74901	9920	Kelch repeat and BTB (POZ) domain containing 11
140.2596	−1.8406	0.0112	0	ILMN_1217776	Mll5	69188	55904	Lysine (K)-specific methyltransferase 2E
123.3913	−1.8632	0.0097	0	ILMN_2466797	scl0001284.1_18	NA	NA	NA
120.66	−1.8657	0.0092	0	ILMN_2495555	Mapk8ip2	NA	NA	NA
110.3349	−1.8716	0.008	0	ILMN_2720479	Lpgat1	226856	9926	Lysophosphatidylglycerol acyltransferase 1
107.1799	−1.8815	0.0084	0	ILMN_3031781	Arid5b	71371	84159	AT-rich interactive domain 5B (MRF1-like)
108.2637	−1.8822	0.0082	0	ILMN_2968123	Slc7a14	241919	57709	Solute carrier family 7 (cationic amino acid transporter, y+ system), member 14
102.8964	−1.8896	0.0082	0	ILMN_2524100	Zswim6	67263	57688	Zinc finger SWIM-type containing 6
106.148	−1.8943	0.008	0	ILMN_1243996	Ash1l	192195	55870	ash1 (absent, small, or homeotic)-like (Drosophila)
108.9677	−1.8986	0.0082	0	ILMN_1226085	Syt1	NA	NA	NA
99.6837	−1.8993	0.0081	0	ILMN_2844963	Nos1ap	70729	NA	Nitric oxide synthase 1 (neuronal) adaptor protein
80.4955	−1.9004	0.0043	0	ILMN_3107059	Espn	56226	83715	Espin
150.8775	−1.9026	0.0136	0	ILMN_1245389	LOC236604	236604	NA	Phosphatidylserine decarboxylase, pseudogene 1
99.2247	−1.9029	0.0085	0	ILMN_2715848	Slitrk4	245446	139065	SLIT and NTRK-like family, member 4
104.8752	−1.9033	0.0083	0	ILMN_2771709	Ppargc1b	170826	133522	Peroxisome proliferative-activated receptor, γ, coactivator 1 β
93.2724	−1.922	0.0076	0	ILMN_3028837	Pcdh9	211712	5101	Protocadherin 9
70.0507	−1.9234	0.0043	0	ILMN_2752883	Hsp90aa1	15519	3320	Heat shock protein 90, alpha (cytosolic), class A member 1
68.4948	−1.9249	0.0045	0	ILMN_2702303	Ch25h	12642	9023	Cholesterol 25-hydroxylase
78.6377	−1.9335	0.0045	0	ILMN_3144164	Irs2	384783	8660	Insulin receptor substrate 2
83.4984	−1.9539	0.005	0	ILMN_1255854	Mtap9	213582	79884	Microtubule-associated protein 9
62.4352	−1.9916	0.0039	0	ILMN_2639442	Rock2	19878	9475	Rho-associated coiled-coil containing protein kinase 2
65.2953	−2.006	0.0047	0	ILMN_2703913	Mtf2	17765	22823	Metal response element binding transcription factor 2
57.2834	−2.0145	0.0018	0	ILMN_1236844	A830094I09Rik	NA	NA	NA
55.3183	−2.0239	0.0021	0	ILMN_1236820	9430047F21Rik	NA	NA	NA
55.866	−2.0392	0.0019	0	ILMN_2432200	Epb4.1	NA	NA	NA
49.8425	−2.0396	0.0023	0	ILMN_2642426	Agmat	75986	79814	agmatine ureohydrolase (agmatinase)
45.5522	−2.0743	0.0017	0	ILMN_1237548	5830407P18Rik	NA	NA	NA
44.5367	−2.0756	0.0018	0	ILMN_1259747	Il33	77125	90865	Interleukin-33
40.11	−2.0942	0.001	0	ILMN_3124885	Pdpk1	18607	5170	3-Phosphoinositide dependent protein kinase 1
35.7678	−2.1106	0	0	ILMN_2601176	Meg3	17263	NA	Maternally expressed 3
38.3554	−2.1249	0.0011	0	ILMN_1225423	Mslnl	328783	NA	Mesothelin-like
55.718	−2.1295	0.002	0	ILMN_2668333	Prg4	96875	NA	Proteoglycan 4 (megakaryocyte stimulating factor, articular superficial zone protein)
27.1219	−2.1716	0	0	ILMN_1216085	B230387C07Rik	106585	23253	Ankyrin repeat domain 12
25.1926	−2.2119	0	0	ILMN_1238436	Cplx2	12890	10814	Complexin 2
107.5742	−2.2202	0.0081	0	ILMN_2965669	Xlr4a	NA	NA	NA
28.7047	−2.2624	0	0	ILMN_2661820	Agxt2l1	71760	64850	Ethanolamine phosphate phospholyase
23.2317	−2.4004	0	0	ILMN_2652500	Lrg1	76905	116844	Leucine-rich alpha-2-glycoprotein 1
11.5889	−2.5107	0	0	ILMN_1229990	Agxt2l1	71760	64850	Ethanolamine phosphate phospholyase
14.3442	−2.7824	0	0	ILMN_2710905	S100a8	20201	6279	S100 calcium binding protein A8 (calgranulin A)
3.6486	−3.7411	0	0	ILMN_2712075	Lcn2	16819	3934	Lipocalin 2

V1 transcriptomes were profiled with microarray from juvenile mice with naturally elevated experience-dependent plasticity to generate the juvenile plasticity differential expression signature. Using RankProd differential expression (DE) analysis, V1 of juvenile male mice C57BL/6 at P29 was compared to adult C57BL/6 mice (>P60; *n* = 3 each group) to identify 248 DE probes, which mapped to 193 unique mouse Entrez IDs. For downstream analysis, mouse Entrez IDs were mapped to human orthologs using the Mouse Genome Informatics homology reference to yield a 176 gene juvenile plasticity signature. FC, fold change. RP, rank product. pfp, percent false positive (i.e. false discovery rate).

### Disease Leverage Analysis (DLA) identifies inflammatory processes as putative disruptors of plasticity

We sought to identify shared pathophysiology across the diverse list of diseases predicted to dysregulate plasticity signatures. To do so, we developed DLA. This approach calculates the association between diseases that dysregulate the plasticity signature genes and the 50 gene sets in the hallmark library (Subramanian et al., 2005) that represent well defined and distinct biological pathways. Specifically, DLA computes a linear regression between the molecular match score (a measure of strength of association between disease and plasticity signatures) and the pathology score (a measure of activity of the biological pathway in a given disease). Large regression coefficients indicate that a given biological pathway is highly active in diseases that dysregulate plasticity gene signatures and may be pathological to developmental plasticity. Using a multiple test-corrected, empirical *p* value threshold of *p* < 5 × 10^−5^, we found that 7 of 14 largest DLA associations were inflammation-related gene sets and that every inflammation-related gene set in the hallmark library was strongly associated with diseases that dysregulate the plasticity signature (7 of 7 inflammation gene sets at a threshold of *p*_corrected_ < 5 × 10^−5^: OR = 25.8, 95% CI = 1.4–491.2, *p* = 2.7 × 10^−3^_a_, Fisher’s exact test; Figs. 1c, 1–1). Moreover, two of these gene sets, tumor necrosis factor-α (TNF-α) signaling via nuclear factor-κB (NF-κB) and interferon-γ (IFN-γ) response, reflect pathways involved in critical period plasticity (Kaneko et al., 2008; Nagakura et al., 2014).

**Figure 1-1.**
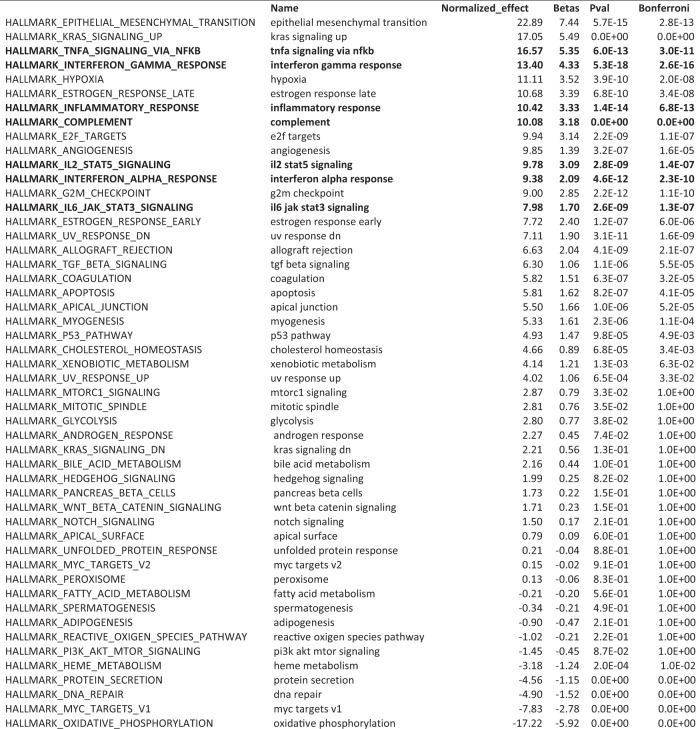
DLA identifies biological processes common to diseases that perturb the juvenile plasticity signature. To identify shared pathophysiology across the diverse list of diseases predicted to dysregulate the juvenile plasticity signature, we applied DLA. This approach calculates the association between diseases that dysregulate the plasticity signature genes and 50 well defined and distinct biological pathways. To do so, it computes a regression between the molecular match score (a measure that indicates the strength of association between the disease and plasticity signatures; see Table 3 and Materials and Methods) and the pathology score (a measure of activity of the biological pathway in that disease; see Materials and Methods). Large regression coefficients indicate that the biological pathway may disrupt juvenile plasticity. Using a multiple test-corrected, empirical *p* < 5 × 10^−5^, seven of the 14 largest DLA associations were inflammation-related gene sets, and every inflammation-related gene set in the hallmark library was strongly associated with diseases that dysregulate plasticity genes (seven of seven inflammation gene sets at *p*_corrected_ < 5 × 10^−5^: OR = 25.8, 95% CI = 1.4–491.2, *p* = 2.7 × 10^−3^, Fisher’s exact test). A total of 20,000 permutations of the gene sets was used to estimate *p* values and to normalize the regression coefficients to allow comparison between effect sizes for different biological pathways. Inflammation-related gene sets: TNF-α signaling via NF-κB, IFN-γ response, inflammatory response, complement, IL-2–Stat5 signaling, IFN-α response, IL-6–Jak–Stat3 signaling.

To control for nonplasticity aspects of age, we repeated the entire analysis using a *Lynx1*^−/−^ plasticity signature of 98 genes, which was identified by computing the differential expression between adult *Lynx1*^−/−^ and adult wild-type V1 (Table 4). By releasing the *Lynx1* brake on plasticity, *Lynx1*^−/−^ mice have juvenile-like plasticity in adulthood (Morishita et al., 2010; Bukhari et al., 2015). Indeed, functional similarity is reflected in signature similarity, as juvenile and *Lynx1*^−/−^ plasticity signatures significantly overlap (35 genes shared, OR = 37.1, 95% CI = 23.8–58.0, *p* < 2.2 × 10^−16^_b_). Applying DLA to the *Lynx1*^−/−^ molecular matches, we found a strong association between diseases predicted to disrupt *Lynx1*^−/−^ plasticity and inflammation-related gene sets (7 of 7 inflammation gene sets at a threshold of *p*_corrected_ < 5 × 10^−5^: OR = 63.0, 95% CI = 3.2–1229.8, *p* = 7.6 × 10^−5^_c_ Fisher’s exact test; Figs. 2, 2-1). Together, the bioinformatics analyses indicate that inflammation is a process central to diseases predicted to dysregulate plasticity gene expression, suggesting that inflammation may disrupt developmental plasticity.

**Figure 2.**
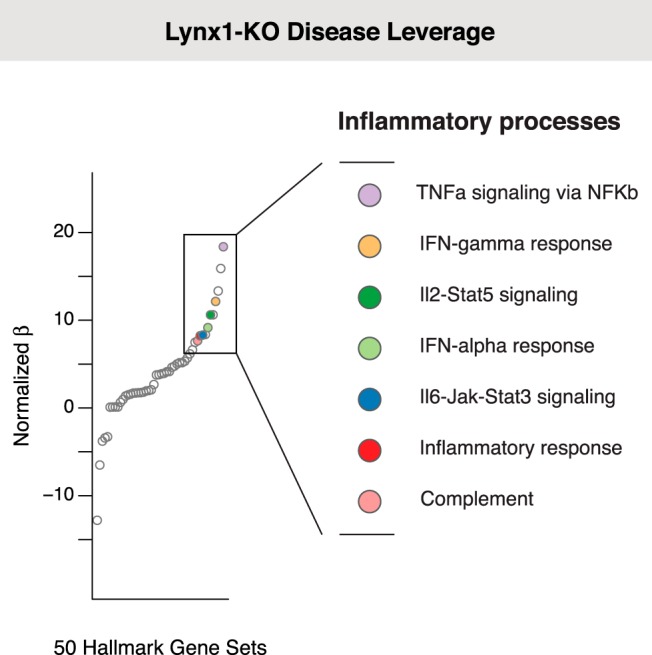
Diseases that dysregulate the adult *Lynx1*^−/−^ plasticity signature are associated with inflammatory processes. Using an adult *Lynx1*^−/−^ plasticity signature of 98 genes (generated by differential expression of primary visual cortex from >P60 *Lynx1*^−/−^ vs >P60 C57BL/6 male mice), DLA systematically identified biological processes associated with diseases that dysregulate the adult *Lynx1*^−/−^ plasticity signature genes. Using adult *Lynx1*^−/−^ animals controls for age, as these adult animals have elevated plasticity similar to that of juvenile animals. Seven of the 11 largest associations were inflammation-related gene sets, and every inflammation-related gene set was strongly associated with plasticity (7 of 7 inflammation gene sets at *p*_corrected_ < 5 × 10^−5^: OR = 63.0, 95% CI = 3.2–1229.8, *p* = 7.6 × 10^−5^, Fisher’s exact test). See Figure 2-1 for source DLA data.

**Figure 2-1.**
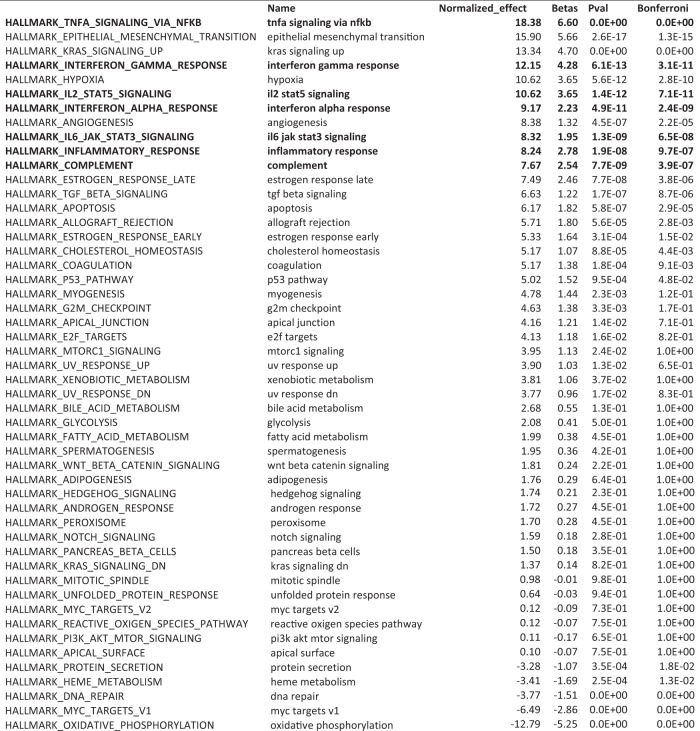
DLA identifies biological processes common to diseases that perturb the *Lynx1*^−/−^ plasticity signature. To identify shared pathophysiology across the diverse list of diseases predicted to dysregulate the *Lynx1*^−/−^ plasticity signature, we applied DLA. Large regression coefficients indicate that the biological pathway may disrupt *Lynx1*^−/−^ plasticity. As with the juvenile plasticity signature, using a multiple test-corrected, empirical *p* < 5 × 10^−5^, we found that every inflammation-related gene set in the hallmark library was strongly associated with diseases that dysregulate plasticity genes (7 of 7 inflammation gene sets at *p*_corrected_ < 5 × 10^−5^: OR = 63.0, 95% CI = 3.2–1229.8, *p* = 7.6 × 10^−5^ Fisher’s exact test). A total of 20,000 permutations of the gene sets were used to estimate *p* values and to normalize the regression coefficients to allow comparison between effect sizes for different biological pathways. Inflammation-related gene sets: TNF-α signaling via NF-κB, IFN-γ response, inflammatory response, complement, IL-2–Stat5 signaling, IFN-α response, IL-6–Jak–Stat3 signaling.

### Lipopolysaccharide model of inflammation suppresses developmental cortical plasticity

**Figure 3.**
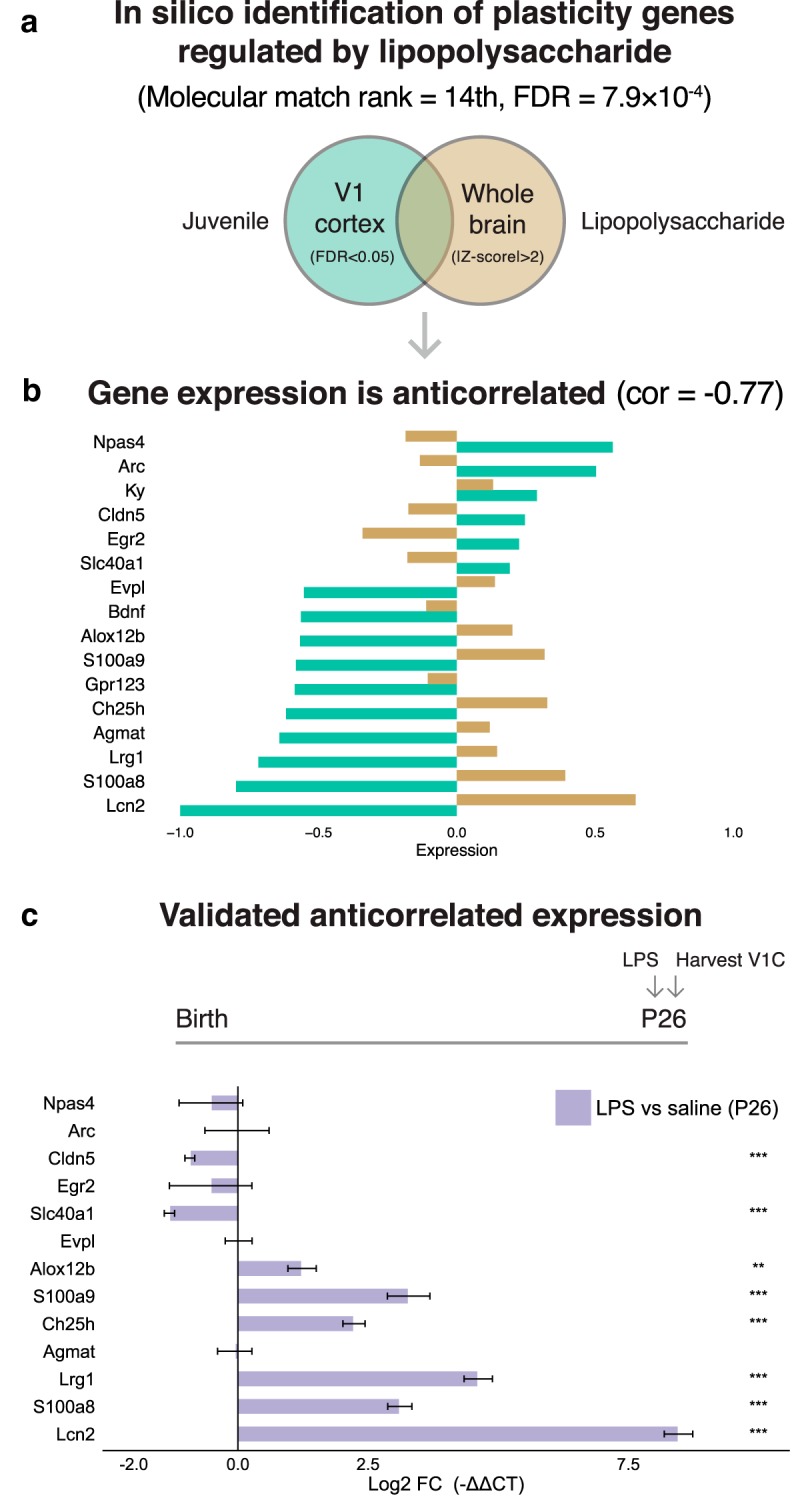
LPS reverses plasticity signature gene expression. ***a***, LPS disease signature shares plasticity signature genes *in silico* (molecular match rank #14, *p* = 7.9 × 10^−4^; Table 3; disease signature is from GSE3253: adult mouse whole-brain homogenate harvested 4 h after peripheral LPS injected intraperitoneally; genes with absolute value of the standardized expression (*z*-score) ≥2 SDs from the mean were selected as the most differentially expressed by LPS). ***b***, The expression of the 16 genes shared between juvenile plasticity and the LPS disease signatures is anticorrelated (Spearman’s ρ = −0.77, *p* = 7.4 × 10^−4^; LPS disease signature gene expression values fell in the range [−1,+1]; for plotting purposes, the plasticity gene expression fold change was linear transformed to [−1,+1]). ***c***, Of the 13 genes of the 16 predicted to be reversed by LPS, the majority (8 of 13; 61.5%) showed a complete reversal in their differential expression pattern in V1 after LPS administration (300 μg/kg LPS, intraperitoneally, at P26 during the peak of juvenile plasticity) relative to saline. LPS downregulated *Cldn5* and *Slc40a1* and upregulated *Alox12b*, *S100a9a*, *Ch25h*, *Lrg1*, *S100a8*, and *Lcn2* (*n* = 5 mice per group). ***p < 0.001, **p > 0.001 and ≤0.01, *p > 0.01 and ≤0.05 (two-sided *t* tests of ΔCTs). Log2 fold change is −ΔΔ CT. Error bars indicate the SEM.

**Figure 4.**
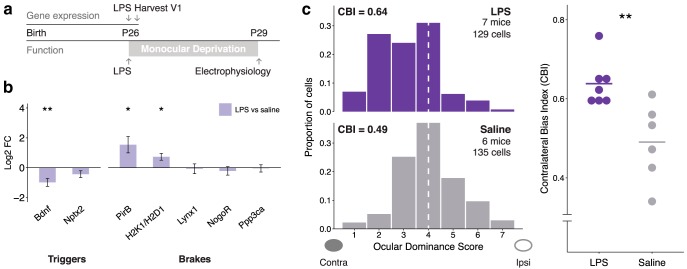
Inflammation induced by LPS suppresses experience-dependent plasticity in juvenile cortex. ***a***, Juvenile mice (P26) during the peak of ocular dominance plasticity were injected intraperitoneally with either LPS (300 μg/kg) or saline. ***b***, ***c***, Mice were either subjected to qPCR analysis of plasticity effectors from V1 4 h after the injection (***b***) or underwent 3 d of MD followed by *in vivo* extracellular recordings to assess ocular dominance plasticity (***c***). ***b***, LPS increased known plasticity brakes *PirB* and *H2K1*/*H2D1*, and decreased the plasticity trigger *BDNF*. LPS had no effect on the plasticity effectors *Nptx2*, *Lynx1*, *NogoR*, or *Ppp3ca*. Log2 FC (fold change) is −ΔΔ CT (*n* = 5 mice per group). Error bar indicates the SEM. ***p < 0.001, **p > 0.001 and ≤0.01, *p > 0.01 and ≤0.05 (*t* test of Δ CTs). ***c***, Neurons from peripheral LPS-treated mice (purple histogram: *n* = 7 mice, 129 cells) showed decreased cortical responsivity to light in the ipsilateral vs contralateral eye, as quantified by a reduced right shift in the ODS distribution after 3 d of MD compared with control saline-treated juvenile mice with MD (gray histogram: 6 mice, 135 cells; χ^2^ test of ODS distribution: *p* = 2.6 × 10^−6^). Animal-level quantification of ocular dominance plasticity by CBI reflects the extent of the ocular dominance shift after 3 d of MD (right-side plot; low CBI indicates higher plasticity). CBI was strongly increased in the LPS-treated group (purple discs: CBI = 0.64 ± 0.02, 7 mice) compared with saline-treated group (gray discs: CBI = 0.49 ± 0.04, 6 mice), indicating that proinflammatory LPS had suppressed developmental plasticity (LPS vs saline, **p = 6 × 10^−3^, one-sided *t* test). qPCR data are reported as the mean ± SEM. Horizontal bars for the CBI plot indicate the mean.

Based on the DLA findings, we hypothesized that inflammatory processes disrupt developmental cortical plasticity. To test this hypothesis, we induced a systemic inflammatory response via LPS and measured the impact on developmental plasticity and related gene expression. We injected a low dose of LPS (300 μg/kg, intraperitoneal) at the peak of juvenile ocular dominance plasticity at P26 and found a strong inflammatory response in V1, as indicated by a 2.4 log_2_ fold increase of interleukin (IL)-1β compared with vehicle control (*p* = 3.4 × 10^−4^_d_, *t* test of Δ CTs, *n* = 5 mice/group). To identify a focused subset of plasticity genes likely to be regulated by LPS (regardless of age or specific brain region) to test *in vivo*, we investigated a highly significant molecular match between the juvenile plasticity signature and an adult brain-derived LPS transcriptome (GSE3253; rank #14, *p* = 7.9 × 10^−4^_e_; empirical *p* value calculated using molecular match algorithm; Fig. 3a; Table 3). Next, we identified a subset of genes from GSE3253 that is likely to play a larger role in the under-lying biology (the genes in the extremes using a *z*-score cutoff) and intersected it with the plasticity signature to identify 16 shared genes (Fig. 3b). Notably, among these shared genes, we identified a negative correlation in their expression pattern (cor = −0.77, *p* = 0.0007_f_, Spearman’s correlation). Among these 16 shared genes, the adult LPS data indicated the direction of expression of 13 genes would be reversed by LPS. Indeed, the majority (61.5%) of the 13 genes showed a complete reversal in their differential expression pattern in V1 after peripheral LPS administration during the critical period (qPCR, all reversed genes *p* < 5 × 10^−4^_g_, *t* test of Δ CTs, *n* = 5 mice per group; Fig. 3c). These data indicate that genes in the plasticity signature that are also regulated by LPS act in an antagonistic fashion and naturally led us to the hypothesis that inflammation may suppress plasticity. Consistent with this logic, the established brakes of plasticity, *PirB* (Syken et al., 2006) and *H2K1* and *H2D1* (Datwani et al., 2009), showed increased expression compared with vehicle (*p* = 0.04_h_ and *p* = 0.03_i_, respectively, *t* test of Δ CTs, *n* = 5 mice per group; Fig. 4a,b). In addition, a trigger of plasticity that increases across development, BDNF (Huang et al., 1999), which we predicted *in silico* to decrease after LPS (Fig. 3b), showed decreased expression compared with vehicle (*p* = 0.009_j_, *t* test of ΔCTs, *n* = 5 mice/group; Fig. 4b). In contrast, other known plasticity effectors (Takesian and Hensch, 2013) were not changed relative to vehicle (*Nptx2*, *Lynx1*, *NogoR*, *Ppp3ca*; *p* > 0.1_k_, *t* test of Δ CTs, *n* = 5 mice per group), indicating that LPS may act through a specific subset of known and novel plasticity effectors (Fig. 4b).

**Table 3:** Molecular matching among 436 disease signatures and the juvenile plasticity signature indicates diverse diseases may disrupt plasticity

Rank	Normalized molecular match score	Emp_pval	FDR	Source_dat	Species	Disease	Tissue
1	9.64	4.53E-08	1.22E-05	GSE7958	*Mus musculus*	Huntington's disease	CNS, brain, striatum (MMHCC)
2	8.35	7.74E-08	1.22E-05	GSE1623	*Mus musculus*	Type 1 diabetes mellitus	Pancreas
3	8.32	8.58E-07	3.40E-05	GSE9857	*Mus musculus*	Huntington's disease	CNS, brain, striatum (MMHCC)
4	7.96	1.38E-07	1.22E-05	GSE4648	*Mus musculus*	Acute myocardial infarction	Myocardial tissue
5	7.59	1.40E-07	1.22E-05	GSE4290	*Homo sapiens*	Glioblastoma	CNS, brain (MMHCC)
6	7.52	3.36E-07	2.44E-05	GSE1481	*Homo sapiens*	Anterior horn cell disease	CNS, spinal cord (MMHCC)
7	6.81	9.04E-08	1.22E-05	GSE2240	*Homo sapiens*	Atrial fibrillation	Myocardial tissue
8	6.73	1.47E-06	4.94E-05	GSE5500	*Mus musculus*	Cardiac hypertrophy	Myocardial tissue
9	6.47	8.10E-07	3.40E-05	GSE5389	*Homo sapiens*	Bipolar disorder	frontal cortex
10	6.33	8.15E-07	3.40E-05	GSE5078	*Mus musculus*	Senescence	Hippocampus
11	6.25	1.75E-06	5.46E-05	GSE4764	*Mus musculus*	*Yersinia enterocolitica* food poisoning	Peyer's patch
12	5.75	7.82E-07	3.40E-05	GSE4290	*Homo sapiens*	Astrocytoma	CNS, brain (MMHCC)
13	5.7	1.73E-05	3.59E-04	GSE1621	*Mus musculus*	Cardiac hypertrophy	Myocardial tissue
14	5.67	6.55E-05	7.88E-04	GSE3253	*Mus musculus*	Bacterial infection	CNS, brain (MMHCC)
15	5.61	1.16E-06	4.22E-05	GSE10599	*Mus musculus*	Spinal muscular atrophy	Kidney
16	5.55	2.49E-06	7.25E-05	GSE3744	*Homo sapiens*	Breast cancer	Mammary gland tissue
17	5.43	1.32E-05	3.04E-04	GSE6731	*Homo sapiens*	Crohn's disease	Intestine, large intestine, colon (MMHCC)
18	5.38	2.24E-05	4.24E-04	GSE6731	*Homo sapiens*	Ulcerative colitis	Intestine, large intestine, colon (MMHCC)
19	5.35	1.29E-05	3.04E-04	GSE11343	*Mus musculus*	Diabetic neuropathy	Sciatic nerve
20	5.35	7.33E-07	3.40E-05	GSE4316	*Homo sapiens*	Primary open angle glaucoma	Trabecular meshwork
21	5.28	2.67E-05	4.65E-04	GSE5406	*Homo sapiens*	Cardiomyopathy	Myocardial tissue
22	5.02	3.36E-05	5.29E-04	GSE464	*Rattus norvegicus*	Spinal cord injury	CNS, spinal cord (MMHCC)
23	4.98	1.87E-05	3.71E-04	EXPE-MEXP-567	*Homo sapiens*	Glioblastoma	Brain
24	4.94	1.17E-04	1.13E-03	GSE1025	*Mus musculus*	DMD	Muscle, striated (skeletal) (MMHCC)
25	4.9	5.26E-05	6.95E-04	GSE1843	*Rattus norvegicus*	Hepatic cirrhosis	Hepatic tissue
26	4.86	1.54E-05	3.37E-04	GSE1572	*Homo sapiens*	Senescence	frontal cortex
27	4.81	6.87E-05	7.88E-04	GSE1472	*Mus musculus*	DMD	Muscle, striated (skeletal) (MMHCC)
28	4.81	9.47E-06	2.43E-04	GSE2510	*Homo sapiens*	Obesity	Adipocyte
29	4.8	3.91E-05	5.49E-04	GSE3383	*Mus musculus*	Cardiac hypertrophy	Myocardial tissue
30	4.78	1.06E-04	1.05E-03	GSE1629	*Homo sapiens*	Dental cavity, complex	Dental pulp
31	4.72	6.79E-05	7.88E-04	GSE2629	*Mus musculus*	Muscular dystrophy	Muscle, striated (skeletal) (MMHCC)
32	4.67	3.90E-05	5.49E-04	GSE4290	*Homo sapiens*	Oligodendroglioma	CNS, brain (MMHCC)
33	4.63	1.71E-04	1.49E-03	GSE10760	*Homo sapiens*	Muscular dystrophy	Musculus vastus lateralis
34	4.62	9.53E-05	1.01E-03	GSE1988	*Mus musculus*	Cardiac failure	Myocardial tissue
35	4.61	4.84E-05	6.59E-04	GSE3467	*Homo sapiens*	Papillary carcinoma of the thyroid	Thyroid gland (MMHCC)
36	4.57	3.84E-05	5.49E-04	GSE3621	*Mus musculus*	Huntington's disease	Brain
37	4.56	3.35E-05	5.29E-04	GSE4107	*Homo sapiens*	Cancer of colon	Intestine, large intestine, colon (MMHCC)
38	4.54	1.20E-04	1.14E-03	GSE842	*Mus musculus*	MS	CNS, spinal cord (MMHCC)
39	4.53	8.18E-05	9.14E-04	GSE14905	*Homo sapiens*	Psoriasis vulgaris	Skin tissue
40	4.51	3.40E-05	5.29E-04	GSE7305	*Homo sapiens*	Endometriosis	Endometrium
41	4.48	8.91E-06	2.43E-04	GSE16524	*Homo sapiens*	Setleis syndrome	Skin fibroblast
42	4.46	6.85E-05	7.88E-04	GSE4183	*Homo sapiens*	Carcinoma *in situ* of large intestine	Colon
43	4.43	2.37E-05	4.30E-04	GSE12654	*Homo sapiens*	Schizophrenia	Cerebral cortex
44	4.3	1.62E-04	1.44E-03	GSE2866	*Mus musculus*	γ-Hydroxybutyric acidemia	CNS, brain, cerebellum (MMHCC)
45	4.22	9.23E-05	1.01E-03	GSE1859	*Mus musculus*	FDIU	Hepatic tissue
46	4.19	5.52E-05	7.08E-04	GSE4587	*Homo sapiens*	Malignant melanoma	Epidermis
47	4.16	2.13E-04	1.82E-03	GSE63	*Mus musculus*	Cerebral infarction	CNS, brain (MMHCC)
48	4.16	2.26E-04	1.83E-03	GSE6614	*Mus musculus*	Generalized seizures	Brain
49	4.08	1.58E-04	1.44E-03	GSE6280	*Homo sapiens*	Chronic kidney disease	Kidney
50	4.01	3.19E-04	2.44E-03	GSE5390	*Homo sapiens*	Down’s syndrome	Brain
51	4.01	2.66E-04	2.11E-03	GSE2052	*Homo sapiens*	Idiopathic fibrosing alveolitis	Lung tissue
52	4	2.23E-04	1.83E-03	GSE1873	*Mus musculus*	Obstructive sleep apnea	Hepatic tissue
53	3.97	4.39E-04	3.07E-03	GSE4183	*Homo sapiens*	IBD	Colon
54	3.96	1.01E-04	1.02E-03	GSE3075	*Mus musculus*	Spinal muscular atrophy, infantile	CNS, spinal cord (MMHCC)
55	3.94	1.42E-04	1.32E-03	GSE3167	*Homo sapiens*	Urothelial carcinoma *in situ*	Urothelium
56	3.93	2.20E-04	1.83E-03	GSE8514	*Homo sapiens*	Adenoma	Adrenal gland
57	3.93	4.01E-04	2.92E-03	GSE6764	*Homo sapiens*	Carcinoma, hepatocellular	Liver
58	3.92	4.89E-04	3.18E-03	GSE3585	*Homo sapiens*	Cardiomyopathy, dilated	Myocardial tissue
59	3.89	3.39E-04	2.55E-03	GSE1420	*Homo sapiens*	Barrett esophagus	Esophageal tissue
60	3.88	9.89E-05	1.02E-03	GSE1420	*Homo sapiens*	Adenocarcinoma of esophagus	Esophageal tissue
61	3.87	4.40E-04	3.07E-03	GSE781	*Homo sapiens*	Clear cell carcinoma of kidney	Renal tissue
62	3.87	5.95E-04	3.51E-03	GSE2528	*Mus musculus*	Breast cancer	Mammary gland
63	3.84	9.37E-04	4.92E-03	GSE1026	*Mus musculus*	DMD	Muscle, striated (skeletal), diaphragm (MMHCC)
64	3.79	5.25E-04	3.35E-03	GSE3678	*Homo sapiens*	Papillary carcinoma of the thyroid	Thyroid gland (MMHCC)
65	3.74	4.88E-04	3.18E-03	GSE4619	*Homo sapiens*	MDS	Bone marrow stem cell
66	3.73	5.92E-04	3.51E-03	GSE3112	*Homo sapiens*	Polymyositis	Muscle tissue
67	3.72	3.51E-04	2.59E-03	GSE3189	*Homo sapiens*	Multiple benign melanocytic nevi	Epidermis
68	3.68	5.76E-04	3.49E-03	GSE769	*Mus musculus*	Cystic fibrosis	Pancreas
69	3.66	5.76E-04	3.49E-03	GSE10432	*Homo sapiens*	Acne	Sebocyte
70	3.65	1.30E-03	6.16E-03	GSE3252	*Mus musculus*	Muscular dystrophy	Muscle, striated (skeletal), diaphragm (MMHCC)
71	3.62	4.81E-04	3.18E-03	GSE12649	*Homo sapiens*	Schizophrenia	Cerebral cortex
72	3.6	7.41E-04	4.17E-03	GSE2501	*Mus musculus*	Thymic carcinoma	Thymus
73	3.59	1.20E-03	5.87E-03	GSE3268	*Homo sapiens*	Squamous cell carcinoma of lung	Lung tissue
74	3.58	7.82E-04	4.32E-03	GSE857	*Mus musculus*	Huntington's disease	CNS, brain (MMHCC)
75	3.58	2.96E-04	2.30E-03	GSE1009	*Homo sapiens*	Diabetic nephropathy	Renal tissue
76	3.57	1.20E-03	5.87E-03	GSE977	*Mus musculus*	Alexander disease	CNS, brain, olfactory bulb (MMHCC)
77	3.55	5.37E-04	3.35E-03	GSE2826	*Mus musculus*	Bruton's agammaglobulinemia	B-cell lymphocyte
78	3.54	7.46E-04	4.17E-03	GSE2640	*Mus musculus*	Pulmonary fibrosis	Lung tissue
79	3.53	5.37E-04	3.35E-03	GSE2514	*Homo sapiens*	Adenocarcinoma of lung	Lung tissue
80	3.53	8.90E-04	4.73E-03	GSE1551	*Homo sapiens*	Dermatomyositis	Muscle, striated (skeletal) (MMHCC)
81	3.52	4.50E-04	3.07E-03	GSE2507	*Mus musculus*	Muscular dystrophy	Muscle, striated (skeletal) (MMHCC)
82	3.5	7.15E-04	4.10E-03	GSE3189	*Homo sapiens*	Malignant melanoma	Epidermis
83	3.5	1.10E-03	5.58E-03	GSE4764	*Mus musculus*	*Y. enterocolitica* food poisoning	Lymph node
84	3.45	8.68E-04	4.67E-03	EXPE-MEXP-567	*Homo sapiens*	Glioblastoma	Brain
85	3.43	4.44E-04	3.07E-03	GSE3408	*Homo sapiens*	Atypical mycobacterial infection	macrophage
86	3.43	1.10E-03	5.58E-03	GSE6428	*Rattus norvegicus*	Type 2 diabetes mellitus	Endocrine pancreas, islet cell of Langerhans, β-cell (MMHCC)
87	3.42	8.45E-04	4.61E-03	GSE10575	*Homo sapiens*	Arthropathy	Chondrocyte
88	3.4	6.73E-04	3.91E-03	GSE9375	*Mus musculus*	Huntington's disease	CNS, brain, striatum (MMHCC)
89	3.39	2.10E-03	9.16E-03	GSE4390	*Mus musculus*	ALS	CNS, spinal cord (MMHCC)
90	3.38	1.21E-03	5.87E-03	GSE3644	*Mus musculus*	Acute pancreatitis	Pancreas
91	3.38	1.30E-03	6.16E-03	GSE3112	*Homo sapiens*	Inclusion body myositides	Muscle tissue
92	3.33	1.10E-03	5.58E-03	GSE466	*Mus musculus*	DMD	Muscle, striated (skeletal) (MMHCC)
93	3.29	1.40E-03	6.36E-03	GSE2144	*Homo sapiens*	GERD	Esophageal tissue
94	3.27	1.40E-03	6.36E-03	EXPE-MEXP-1296	*Mus musculus*	Cancer of prostate	Prostate
95	3.24	1.70E-03	7.64E-03	GSE2724	*Homo sapiens*	Uterine leiomyoma	Uterus
96	3.21	3.30E-03	1.30E-02	GSE2825	*Rattus norvegicus*	Idiosyncratic drug effect	Hepatic tissue
97	3.19	2.60E-03	1.08E-02	GSE1007	*Homo sapiens*	DMD	Muscle, striated (skeletal) (MMHCC)
98	3.19	1.20E-03	5.87E-03	GSE1781	*Rattus norvegicus*	Sepsis	Hepatic tissue
99	3.18	2.40E-03	1.03E-02	GSE2899	*Mus musculus*	Type 2 diabetes mellitus	Adipose tissue
100	3.18	1.90E-03	8.37E-03	GSE2557	*Mus musculus*	Kidney disorder associated with type 2 diabetes mellitus	Mesangial cell
101	3.18	1.40E-03	6.36E-03	GSE3524	*Homo sapiens*	Squamous cell carcinoma of mouth	Oropharynx epithelium
102	3.17	1.40E-03	6.36E-03	GSE13355	*Homo sapiens*	Psoriasis vulgaris	Skin tissue
103	3.15	2.20E-03	9.50E-03	GSE2866	*Mus musculus*	γ-Hydroxybutyric acidemia	Cerebral cortex
104	3.12	2.60E-03	1.08E-02	GSE4911	*Mus musculus*	Cleidocranial dysostosis	Humerus
105	3.12	1.90E-03	8.37E-03	GSE24807	*Homo sapiens*	NASH	Liver
106	3.12	3.20E-03	1.28E-02	GSE10631	*Homo sapiens*	LGLL	Peripheral blood mononuclear cell
107	3.11	2.70E-03	1.11E-02	GSE2052	*Homo sapiens*	Scleroderma	Lung tissue
108	3.07	2.90E-03	1.17E-02	GSE4692	*Mus musculus*	Obesity	Adipose tissue
109	3.05	2.50E-03	1.06E-02	GSE4824	*Homo sapiens*	Non-small cell lung cancer	Bronchial epithelium
110	3.03	3.80E-03	1.47E-02	GSE1145	*Homo sapiens*	Ischemic cardiomyopathy	Myocardial tissue
111	3.01	4.90E-03	1.77E-02	GSE6678	*Mus musculus*	Infantile neuronal ceroid lipofuscinosis	Brain
112	3	3.30E-03	1.30E-02	GSE3064	*Homo sapiens*	Dystonia	Brain
113	2.99	4.70E-03	1.72E-02	GSE2527	*Mus musculus*	Thrombocytopenia	Megakaryocyte
114	2.99	4.30E-03	1.62E-02	GSE2433	*Mus musculus*	Leukemia, acute megakaryocytic	Megakaryocyte
115	2.93	4.60E-03	1.71E-02	GSE1776	*Mus musculus*	Nutritional deficiency, NEC	Skeletal myocyte
116	2.93	3.90E-03	1.49E-02	GSE4587	*Homo sapiens*	In situ melanoma of skin	Epidermis
117	2.92	5.30E-03	1.88E-02	GSE9579	*Homo sapiens*	Appendicitis	Appendix
118	2.91	5.80E-03	2.01E-02	GSE2411	*Mus musculus*	Ventilator-associated lung injury	Lung tissue
119	2.9	2.80E-03	1.14E-02	GSE1037	*Homo sapiens*	Small cell carcinoma of lung	Lung tissue
120	2.86	5.20E-03	1.86E-02	GSE2401	*Rattus norvegicus*	Hypotensive episode	Renal tissue
121	2.85	4.70E-03	1.72E-02	GSE765	*Mus musculus*	Cystic fibrosis	Intestinal epithelium
122	2.83	3.60E-03	1.40E-02	EXPE-MEXP-882	*Homo sapiens*	Invasive ductal breast cancer	Mammary epithelium
123	2.83	6.70E-03	2.28E-02	GSE1869	*Homo sapiens*	Ischemic cardiomyopathy	Myocardial tissue
124	2.82	5.60E-03	1.95E-02	GSE4183	*Homo sapiens*	Adenoma	Colon
125	2.82	5.40E-03	1.90E-02	GSE1004	*Homo sapiens*	DMD	Muscle, striated (skeletal) (MMHCC)
126	2.81	4.30E-03	1.62E-02	GSE9944	*Homo sapiens*	Glaucoma	Astrocyte
127	2.79	6.20E-03	2.13E-02	EXPE-TABM-176	*Homo sapiens*	Irritable bowel syndrome variant of childhood with diarrhea	Sigmoid colon
128	2.72	8.40E-03	2.75E-02	GSE2779	*Homo sapiens*	MDS	Bone marrow stem cell
129	2.72	7.20E-03	2.43E-02	GSE1947	*Mus musculus*	Peripheral motor neuropathy	Sciatic nerve
130	2.71	8.40E-03	2.75E-02	GSE593	*Homo sapiens*	Uterine leiomyoma	Uterus, myometrium (MMHCC)
131	2.71	7.50E-03	2.52E-02	GSE2712	*Homo sapiens*	Clear cell sarcoma of kidney	Renal tissue
132	2.69	8.50E-03	2.75E-02	GSE1459	*Mus musculus*	Bacterial Infection	Macrophage
133	2.67	8.50E-03	2.75E-02	GSE1811	*Mus musculus*	Occupational lung disease	Lung Tissue
134	2.67	8.90E-03	2.85E-02	GSE2508	*Homo sapiens*	Obesity	Adipocyte
135	2.66	8.10E-03	2.70E-02	GSE1462	*Homo sapiens*	MELAS (mitochondrial myopathy, encephalopathy, lactic acidosis and stroke-like episodes)	Muscle, striated (skeletal) (MMHCC)
136	2.65	9.00E-03	2.86E-02	GSE4130	*Rattus norvegicus*	Dehydration	CNS, brain, hypothalamus (MMHCC)
137	2.61	9.10E-03	2.88E-02	GSE1378	*Homo sapiens*	Breast cancer	Mammary gland tissue
138	2.6	1.14E-02	3.45E-02	GSE1375	*Mus musculus*	Alzheimer's disease	Cerebral cortex
139	2.59	9.80E-03	3.03E-02	GSE2719	*Homo sapiens*	Gastrointestinal stromal tumor	Connective tissue
140	2.59	9.60E-03	3.01E-02	GSE7036	*Homo sapiens*	Bipolar disorder	Lymphocyte
141	2.57	1.00E-02	3.07E-02	GSE1724	*Homo sapiens*	Scleroderma	Fibroblast
142	2.56	9.70E-03	3.02E-02	GSE3790	*Homo sapiens*	Huntington's disease	Caudate nucleus
143	2.55	1.25E-02	3.68E-02	GSE6764	*Homo sapiens*	Hepatic cirrhosis	Liver
144	2.54	1.24E-02	3.68E-02	GSE2457	*Rattus norvegicus*	Erectile dysfunction associated with type 2 diabetes mellitus	Penis, NOS
145	2.54	1.47E-02	4.14E-02	GSE2457	*Rattus norvegicus*	Erectile dysfunction	Penis erectile tissue
146	2.54	1.05E-02	3.20E-02	GSE3110	*Rattus norvegicus*	Dehydration	Hypothalamus
147	2.53	1.22E-02	3.64E-02	GSE2712	*Homo sapiens*	Nephroblastoma	Renal tissue
148	2.51	1.42E-02	4.05E-02	GSE7999	*Rattus norvegicus*	Tachycardia	Myocardial tissue
149	2.51	1.29E-02	3.77E-02	GSE609	*Homo sapiens*	SCID	Thymic lymphocyte
150	2.49	1.19E-02	3.58E-02	GSE1869	*Homo sapiens*	Cardiomyopathy	Myocardial tissue
151	2.49	1.36E-02	3.93E-02	GSE7621	*Homo sapiens*	Parkinson's disease	Substantia nigra
152	2.48	1.35E-02	3.92E-02	GSE10971	*Homo sapiens*	Serous carcinoma	Fallopian tube
153	2.48	1.40E-02	4.02E-02	GSE3418	*Mus musculus*	Lung transplant rejection	Trachea
154	2.47	1.45E-02	4.11E-02	EXPE-MEXP-476	*Homo sapiens*	Cancer of prostate	Endothelial cell
155	2.47	1.54E-02	4.28E-02	GSE3184	*Mus musculus*	Asthma, allergic	Lung tissue
156	2.46	1.49E-02	4.16E-02	GSE4616	*Mus musculus*	Type 1 diabetes mellitus	Myocardial tissue
157	2.41	1.68E-02	4.64E-02	GSE4612	*Mus musculus*	Carcinoma, hepatocellular	Hepatic tissue
158	2.4	1.77E-02	4.85E-02	GSE2725	*Homo sapiens*	Uterine leiomyoma	Uterus
159	2.39	1.81E-02	4.90E-02	GSE3365	*Homo sapiens*	Ulcerative colitis	Peripheral blood mononuclear cell
160	2.37	1.80E-02	4.90E-02	GSE1813	*Rattus norvegicus*	Obesity	Adipose tissue
161	2.36	1.87E-02	5.03E-02	GSE4060	*Homo sapiens*	Cushing syndrome	Adrenal gland
162	2.35	2.07E-02	5.50E-02	GSE1145	*Homo sapiens*	Primary cardiomyopathy	Myocardial tissue
163	2.31	1.88E-02	5.03E-02	GSE6461	*Mus musculus*	Synovial sarcoma	Synovial membrane
164	2.3	2.15E-02	5.68E-02	GSE1037	*Homo sapiens*	Non-small cell lung cancer	Lung tissue
165	2.27	2.29E-02	6.01E-02	GSE10921	*Homo sapiens*	Idiopathic fibrosing alveolitis	Fibroblast
166	2.26	2.41E-02	6.28E-02	GSE3248	*Mus musculus*	Huntington's disease	CNS, brain, cerebellum (MMHCC)
167	2.25	2.42E-02	6.28E-02	GSE4788	*Mus musculus*	Parkinson's disease	Substantia nigra
168	2.24	2.52E-02	6.46E-02	GSE4710	*Mus musculus*	Heart injury	Myocardial tissue
169	2.21	2.52E-02	6.46E-02	GSE6364	*Homo sapiens*	Endometriosis	Endometrium
170	2.2	2.68E-02	6.83E-02	GSE1462	*Homo sapiens*	CPEO (chronic progressive external ophthalmoplegia)	Muscle, striated (skeletal) (MMHCC)
171	2.19	3.00E-02	7.56E-02	GSE1724	*Homo sapiens*	Idiopathic fibrosing alveolitis	Fibroblast
172	2.18	2.87E-02	7.28E-02	GSE6955	*Homo sapiens*	Rett syndrome	frontal cortex
173	2.13	3.24E-02	8.12E-02	GSE1017	*Homo sapiens*	Myopathy NOS	Skeletal muscle
174	2.13	3.39E-02	8.45E-02	GSE2429	*Homo sapiens*	Breast cancer	Mammary gland tissue
175	2.12	3.57E-02	8.79E-02	GSE6764	*Homo sapiens*	Hepatocellular dysplasia	Liver
176	2.11	3.64E-02	8.92E-02	GSE4516	*Rattus norvegicus*	Smoke inhalation	Lung tissue
177	2.1	3.41E-02	8.45E-02	GSE567	*Homo sapiens*	Essential thrombocythemia	Megakaryocyte
178	2.09	3.92E-02	9.39E-02	EXPE-TABM-176	*Homo sapiens*	Irritable bowel syndrome variant of childhood with constipation	Sigmoid colon
179	2.08	3.69E-02	8.99E-02	GSE4130	*Rattus norvegicus*	Dehydration	Pituitary gland
180	2.07	3.72E-02	9.01E-02	GSE9006	*Homo sapiens*	Type 2 diabetes mellitus	Peripheral blood mononuclear cell
181	2.06	4.00E-02	9.40E-02	GSE2899	*Mus musculus*	Type 2 diabetes mellitus	Soleus muscle
182	2.06	4.01E-02	9.40E-02	GSE3125	*Rattus norvegicus*	Dehydration	Hypothalamus
183	2.06	3.99E-02	9.40E-02	GSE3407	*Homo sapiens*	Cockayne syndrome	Fibroblast
184	2.06	3.79E-02	9.13E-02	GSE2866	*Mus musculus*	γ-Hydroxybutyric acidemia	Hippocampus
185	2.05	4.07E-02	9.49E-02	GSE2077	*Homo sapiens*	Protozoan Infection	Intestinal epithelium
186	2.05	4.14E-02	9.60E-02	GSE9006	*Homo sapiens*	Type 1 diabetes mellitus	Peripheral blood mononuclear cell
187	2.03	4.01E-02	9.40E-02	GSE10123	*Homo sapiens*	Premature aging	Skin fibroblast
188	2.01	4.45E-02	1.01E-01	GSE3365	*Homo sapiens*	Crohn's disease	Peripheral blood mononuclear cell
189	2.01	4.33E-02	9.99E-02	GSE1297	*Homo sapiens*	Alzheimer's disease	Hippocampus
190	2	4.40E-02	1.01E-01	GSE2254	*Mus musculus*	Type 1 diabetes mellitus	pancreatic islet
191	2	4.57E-02	1.03E-01	GSE3489	*Homo sapiens*	HIV encephalitis	frontal cortex
192	2	4.44E-02	1.01E-01	GSE3203	*Mus musculus*	Influenza	B-cell lymphocyte
193	1.99	4.67E-02	1.05E-01	GSE2559	*Homo sapiens*	Pulmonary hypertension, primary	Pulmonary artery
194	1.98	4.76E-02	1.06E-01	GSE3807	*Homo sapiens*	Aplastic anemia	Bone marrow
195	1.98	4.85E-02	1.07E-01	GSE4105	*Rattus norvegicus*	Myocardial infarction	Myocardial tissue
196	1.96	4.72E-02	1.06E-01	GSE4479	*Mus musculus*	Sepsis	Splenocyte
197	1.95	5.26E-02	1.15E-01	GSE3676	*Mus musculus*	Infertility due to azoospermia	Testicular tissue
198	1.94	5.21E-02	1.15E-01	GSE10064	*Homo sapiens*	MS	B-cell lymphocyte
199	1.93	5.48E-02	1.17E-01	GSE5090	*Homo sapiens*	Polycystic ovary syndrome	Adipose tissue
200	1.93	5.43E-02	1.17E-01	GSE11	*Mus musculus*	Type 1 diabetes mellitus	Thymic epithelial cell
201	1.92	5.28E-02	1.15E-01	GSE582	*Mus musculus*	Transplanted heart complication	Myocardial tissue
202	1.92	5.44E-02	1.17E-01	GSE10586	*Homo sapiens*	Type 1 diabetes mellitus	T lymphocyte
203	1.92	5.77E-02	1.21E-01	GSE2049	*Homo sapiens*	AML	Haematopoietic stem cell
204	1.91	5.56E-02	1.18E-01	GSE11208	*Homo sapiens*	Nicotine addiction	Ganglioneuroblastoma
205	1.91	5.61E-02	1.18E-01	GSE5538	*Mus musculus*	Hepatic lipidosis	Hepatic Tissue
206	1.9	5.37E-02	1.16E-01	EXPE-MEXP-692	*Homo sapiens*	Barrett esophagus	Esophagus
207	1.9	5.60E-02	1.18E-01	GSE1659	*Mus musculus*	Type 1 diabetes mellitus	Muscle, striated (skeletal) (MMHCC)
208	1.89	6.00E-02	1.25E-01	GSE4170	*Homo sapiens*	CML	Haematopoietic stem cell
209	1.88	6.33E-02	1.31E-01	EXPE-MEXP-692	*Homo sapiens*	Adenocarcinoma of esophagus	Esophagus
210	1.86	6.21E-02	1.29E-01	GSE422	*Mus musculus*	Carcinoma *in situ* of small intestine	Intestinal epithelium
211	1.82	6.93E-02	1.41E-01	GSE1822	*Homo sapiens*	Ewing's sarcoma	Renal tissue
212	1.81	6.69E-02	1.37E-01	GSE755	*Homo sapiens*	Osteolysis	Leukocyte, lymphocyte, B lymphocyte, plasma cell (MMHCC)
213	1.81	6.60E-02	1.36E-01	GSE3004	*Homo sapiens*	Asthma, allergic	Bronchial epithelium
214	1.78	7.26E-02	1.47E-01	GSE4060	*Homo sapiens*	ACTH-dependent Cushing syndrome	Adrenal gland
215	1.76	7.71E-02	1.54E-01	GSE4120	*Mus musculus*	Arrhythmogenic right ventricular cardiomyopathy	Myocardial tissue
216	1.76	8.01E-02	1.59E-01	GSE3790	*Homo sapiens*	Huntington's disease	frontal cortex
217	1.76	7.70E-02	1.54E-01	GSE9800	*Homo sapiens*	Cardiomyopathy, dilated	Myocardial tissue
218	1.71	8.68E-02	1.70E-01	GSE3915	*Mus musculus*	Cancer of the intestine	Small bowel
219	1.69	9.30E-02	1.80E-01	GSE5581	*Mus musculus*	Retinoschisis	Retina
220	1.68	8.96E-02	1.75E-01	GSE868	*Mus musculus*	Familial hypophosphatemic rickets	Renal tissue
221	1.68	8.99E-02	1.75E-01	EXPE-TABM-176	*Homo sapiens*	IBS	Sigmoid colon
222	1.66	9.61E-02	1.85E-01	GSE6965	*Homo sapiens*	Infection by *Aspergillus fumigatus*	Dendritic cell
223	1.66	9.71E-02	1.87E-01	GSE6012	*Homo sapiens*	Eczema	Integument
224	1.65	9.80E-02	1.87E-01	GSE3554	*Mus musculus*	Glaucoma	Retina
225	1.65	1.00E-01	1.90E-01	GSE2223	*Homo sapiens*	Glioblastoma	CNS, brain (MMHCC)
226	1.63	9.98E-02	1.90E-01	EXPE-TABM-26	*Homo sapiens*	Cancer of prostate	Prostate
227	1.62	1.03E-01	1.94E-01	GSE4172	*Homo sapiens*	Viral cardiomyopathy	Myocardial tissue
228	1.6	1.08E-01	2.01E-01	GSE2191	*Homo sapiens*	AML	Mononuclear leukocyte
229	1.6	1.08E-01	2.01E-01	GSE2155	*Homo sapiens*	Breast cancer	Epithelial cell
230	1.56	1.18E-01	2.16E-01	GSE1299	*Homo sapiens*	Breast cancer	Mammary epithelium
231	1.55	1.15E-01	2.13E-01	GSE3320	*Homo sapiens*	COPD	Bronchial epithelium
232	1.55	1.19E-01	2.17E-01	GSE5774	*Homo sapiens*	Nonspecific interstitial pneumonia	Lung tissue
233	1.55	1.18E-01	2.16E-01	GSE6710	*Homo sapiens*	Psoriasis vulgaris	Skin tissue
234	1.53	1.29E-01	2.31E-01	GSE4250	*Homo sapiens*	Hereditary gingival fibromatosis	Gingiva
235	1.53	1.24E-01	2.25E-01	GSE5364	*Homo sapiens*	Cancer of thyroid	Thyroid
236	1.53	1.18E-01	2.16E-01	GSE3583	*Mus musculus*	Huntington's disease	CNS, brain, striatum (MMHCC)
237	1.52	1.30E-01	2.32E-01	GSE12654	*Homo sapiens*	Bipolar disorder	Cerebral cortex
238	1.52	1.29E-01	2.31E-01	GSE973	*Homo sapiens*	Funisitis	Umbilical blood
239	1.51	1.32E-01	2.34E-01	GSE12649	*Homo sapiens*	Bipolar disorder	Cerebral cortex
240	1.5	1.33E-01	2.35E-01	GSE925	*Rattus norvegicus*	Cardiac hypertrophy	Cardiomyocyte
241	1.48	1.38E-01	2.42E-01	GSE3544	*Mus musculus*	MODY	Pancreatic islet
242	1.47	1.42E-01	2.46E-01	GSE420	*Homo sapiens*	Atherosclerosis	Aorta smooth muscle tissue
243	1.47	1.37E-01	2.42E-01	GSE422	*Mus musculus*	Adenoma of small intestine	Intestinal epithelium
244	1.46	1.41E-01	2.46E-01	EXPE-TABM-36	*Homo sapiens*	Hepatocellular adenoma	Hepatocyte
245	1.45	1.38E-01	2.42E-01	GSE4302	*Homo sapiens*	Asthma	Epithelial cell
246	1.45	1.44E-01	2.50E-01	GSE5370	*Homo sapiens*	Dermatomyositis	Muscle, striated (skeletal) (MMHCC)
247	1.42	1.53E-01	2.61E-01	GSE2172	*Mus musculus*	Colitis	Intestine, large intestine, colon (MMHCC)
248	1.41	1.55E-01	2.63E-01	GSE11750	*Homo sapiens*	Senescence	Muscle, striated (skeletal) (MMHCC)
249	1.41	1.56E-01	2.63E-01	GSE3	*Homo sapiens*	Chromophobe cell carcinoma of kidney	Renal cell
250	1.41	1.52E-01	2.61E-01	GSE2705	*Homo sapiens*	Primary open angle glaucoma	Optic disk
251	1.37	1.68E-01	2.80E-01	GSE3860	*Homo sapiens*	Progeria	Fibroblast
252	1.37	1.73E-01	2.86E-01	GSE674	*Homo sapiens*	Senescence	Muscle, striated (skeletal) (MMHCC)
253	1.36	1.72E-01	2.84E-01	GSE2549	*Homo sapiens*	Malignant mesothelioma of pleura	Pleura
254	1.36	1.71E-01	2.84E-01	GSE1466	*Homo sapiens*	Lymphoblastic leukemia	T lymphocyte
255	1.33	1.82E-01	2.97E-01	GSE1611	*Mus musculus*	Down syndrome	CNS, brain, cerebellum (MMHCC)
256	1.33	1.79E-01	2.93E-01	GSE997	*Homo sapiens*	Essential thrombocythemia	Megakaryocyte
257	1.32	1.91E-01	3.09E-01	GSE6798	*Homo sapiens*	Polycystic ovary syndrome	Skeletal muscle
258	1.32	1.83E-01	2.98E-01	GSE3790	*Homo sapiens*	Huntington's disease	CNS, brain, cerebellum (MMHCC)
259	1.31	1.87E-01	3.03E-01	GSE2503	*Homo sapiens*	Actinic keratosis	Skin tissue
260	1.29	1.94E-01	3.12E-01	GSE1010	*Homo sapiens*	Familial combined hyperlipidemia	Lymphoblast
261	1.27	2.00E-01	3.17E-01	GSE1028	*Homo sapiens*	Scott syndrome	B-cell lymphocyte
262	1.27	1.98E-01	3.16E-01	GSE5388	*Homo sapiens*	Bipolar disorder	Frontal cortex
263	1.27	2.00E-01	3.17E-01	GSE2067	*Homo sapiens*	Hepatitis C infection	Hepatocyte
264	1.26	2.04E-01	3.22E-01	GSE3280	*Homo sapiens*	Acute monocytic leukemia	Blood monocyte
265	1.26	2.07E-01	3.24E-01	GSE642	*Mus musculus*	Type 2 diabetes mellitus	Renal tissue
266	1.26	2.07E-01	3.24E-01	GSE5281	*Homo sapiens*	Alzheimer's disease	Superior frontal gyrus
267	1.21	2.28E-01	3.54E-01	GSE11348	*Homo sapiens*	Rhinovirus infection	Nose
268	1.2	2.34E-01	3.61E-01	GSE7486	*Homo sapiens*	Epilepsy	Lymphoblast
269	1.16	2.46E-01	3.74E-01	GSE3384	*Mus musculus*	Nemaline myopathy	Tibialis Muscle
270	1.16	2.45E-01	3.73E-01	GSE473	*Homo sapiens*	Asthma	CD4-positive lymphocyte
271	1.16	2.45E-01	3.73E-01	GSE474	*Homo sapiens*	Obesity	Muscle, striated (skeletal) (MMHCC)
272	1.14	2.50E-01	3.78E-01	EXPE-MEXP-669	*NULL*	Neuroblastoma	Adrenal cortex
273	1.12	2.62E-01	3.95E-01	GSE3889	*Mus musculus*	Hypercholesterolemia	Hepatic tissue
274	1.11	2.66E-01	3.97E-01	GSE2175	*Homo sapiens*	Adenoma of pituitary gland	Pituitary gland
275	1.1	2.71E-01	4.03E-01	GSE3925	*Mus musculus*	Hyperreactive airway disease	Lung tissue
276	1.09	2.79E-01	4.13E-01	GSE10162	*Mus musculus*	Nephrolithiasis	Kidney
277	1.09	2.62E-01	3.95E-01	GSE2470	*Rattus norvegicus*	Type 2 diabetes mellitus	Pancreas
278	1.08	2.77E-01	4.10E-01	GSE362	*Homo sapiens*	Senescence	Muscle, striated (skeletal) (MMHCC)
279	1.06	2.87E-01	4.23E-01	GSE2973	*Mus musculus*	Infection by *Y. enterocolitica*	Macrophage
280	1.04	2.98E-01	4.35E-01	GSE4785	*Homo sapiens*	Simian acquired immune deficiency syndrome	T lymphocyte
281	1.04	2.97E-01	4.35E-01	GSE923	*Homo sapiens*	*Pseudomonas* infection	Lung tissue
282	1.02	3.07E-01	4.46E-01	GSE12654	*Homo sapiens*	Depression	Cerebral cortex
283	1.01	3.08E-01	4.47E-01	GSE2053	*Homo sapiens*	RA	Synovial membrane
284	1.01	3.14E-01	4.54E-01	GSE1037	*Homo sapiens*	Adenocarcinoma of lung	Lung tissue
285	1	3.18E-01	4.58E-01	GSE4333	*Mus musculus*	Lymphatic edema	Skin tissue
286	0.99	3.22E-01	4.62E-01	GSE10964	*Mus musculus*	COPD	Lung
287	0.99	3.28E-01	4.69E-01	GSE2657	*Homo sapiens*	Sarcoidosis	T lymphocyte
288	0.96	3.41E-01	4.81E-01	GSE1767	*Homo sapiens*	Huntington's disease	Blood
289	0.96	3.35E-01	4.77E-01	GSE7753	*Homo sapiens*	JRA	Peripheral blood mononuclear cell
290	0.95	3.40E-01	4.81E-01	GSE4125	*Homo sapiens*	Adenocarcinoma of kidney	Kidney
291	0.94	3.46E-01	4.87E-01	GSE8762	*Homo sapiens*	Huntington's disease	Lymphocyte
292	0.93	3.48E-01	4.88E-01	GSE5788	*Homo sapiens*	Leukemia, chronic T cell	T lymphocyte
293	0.92	3.56E-01	4.98E-01	GSE483	*Mus musculus*	Asthma, allergic	Lung tissue
294	0.91	3.58E-01	4.99E-01	GSE2566	*Rattus norvegicus*	Neurogenic muscular atrophy	Muscle, striated (skeletal) (MMHCC)
295	0.91	3.60E-01	4.99E-01	GSE2600	*Homo sapiens*	Anaplasmosis	Leukemic cell
296	0.91	3.68E-01	5.06E-01	GSE2127	*Mus musculus*	Carcinoma, hepatocellular	Hepatic tissue
297	0.91	3.59E-01	4.99E-01	GSE2052	*Homo sapiens*	MCTD	Lung tissue
298	0.84	3.85E-01	5.26E-01	GSE2276	*Mus musculus*	Asthma, allergic	Lung tissue
299	0.84	4.07E-01	5.52E-01	GSE4707	*Homo sapiens*	Preeclampsia	Placenta
300	0.81	4.22E-01	5.68E-01	GSE3871	*Homo sapiens*	Androgen insensitivity syndrome	Fibroblast
301	0.81	4.17E-01	5.63E-01	GSE1606	*Mus musculus*	Turner syndrome	CNS, brain (MMHCC)
302	0.8	4.27E-01	5.70E-01	GSE1674	*Mus musculus*	Hypertension	Adrenal gland
303	0.8	4.28E-01	5.70E-01	GSE3195	*Mus musculus*	Hypoxia	Fibroblast
304	0.79	4.28E-01	5.70E-01	GSE475	*Homo sapiens*	COPD	Muscle, striated (skeletal), diaphragm (MMHCC)
305	0.79	4.34E-01	5.75E-01	GSE1872	*Rattus norvegicus*	Breast cancer	Mammary gland tissue
306	0.79	4.29E-01	5.70E-01	GSE3868	*Homo sapiens*	Cancer of prostate	Prostate
307	0.76	4.46E-01	5.87E-01	EXPE-MEXP-669	*NULL*	Neuroblastoma	Adrenal cortex
308	0.76	4.43E-01	5.85E-01	GSE5109	*Homo sapiens*	Obesity	Muscle, striated (skeletal) (MMHCC)
309	0.73	4.64E-01	6.07E-01	GSE1413	*Mus musculus*	Cancer of prostate	Prostate
310	0.72	4.76E-01	6.18E-01	GSE5281	*Homo sapiens*	Alzheimer's Disease	Middle temporal gyrus
311	0.72	4.71E-01	6.15E-01	GSE3384	*Mus musculus*	Nemaline myopathy	Extensor digitorum longus muscle
312	0.69	4.89E-01	6.32E-01	GSE2223	*Homo sapiens*	Astrocytoma	CNS, brain (MMHCC)
313	0.68	4.97E-01	6.42E-01	EXPE-MEXP-476	*Homo sapiens*	Cancer of prostate	Endothelial cell
314	0.68	4.99E-01	6.42E-01	EXPE-MEXP-476	*Homo sapiens*	Cancer of prostate	Endothelial cell
315	0.66	5.10E-01	6.54E-01	GSE9877	*Homo sapiens*	Sickle cell anemia	Endothelial cell
316	0.65	5.18E-01	6.62E-01	GSE3026	*Homo sapiens*	Bacterial infection	Peripheral blood mononuclear cell
317	0.63	5.30E-01	6.76E-01	GSE128	*Mus musculus*	Retinitis pigmentosa	Retina
318	0.61	5.34E-01	6.77E-01	GSE1379	*Homo sapiens*	Breast cancer	Mammary gland tissue
319	0.58	5.63E-01	7.02E-01	GSE867	*Mus musculus*	Hepatitis, autoimmune	Hepatic tissue
320	0.58	5.63E-01	7.02E-01	GSE465	*Homo sapiens*	DMD	Muscle, striated (skeletal) (MMHCC)
321	0.58	5.61E-01	7.02E-01	GSE1871	*Mus musculus*	Acute lung injury	Lung tissue
322	0.57	5.65E-01	7.02E-01	GSE5900	*Homo sapiens*	MGUS	Bone marrow
323	0.57	5.70E-01	7.02E-01	GSE2223	*Homo sapiens*	Oligodendroglioma	CNS, brain (MMHCC)
324	0.57	5.65E-01	7.02E-01	GSE1055	*Rattus norvegicus*	Cardiac hypertrophy	Cardiomyocyte
325	0.57	5.70E-01	7.02E-01	GSE4748	*Homo sapiens*	Bacterial infection	Dendritic cell
326	0.57	5.73E-01	7.03E-01	GSE3384	*Mus musculus*	Nemaline myopathy	Muscle, striated (skeletal), diaphragm (MMHCC)
327	0.57	5.70E-01	7.02E-01	GSE2236	*Mus musculus*	Congestive heart disease	Myocardial tissue
328	0.56	5.74E-01	7.03E-01	GSE1008	*Mus musculus*	DMD	Extraocular muscle
329	0.53	5.90E-01	7.15E-01	GSE1472	*Mus musculus*	DMD	Extraocular muscle
330	0.53	6.02E-01	7.20E-01	GSE85	*Mus musculus*	APECED	Thymic epithelial cell
331	0.53	5.92E-01	7.15E-01	GSE1849	*Homo sapiens*	Sickle cell anemia	Pulmonary artery
332	0.52	5.99E-01	7.19E-01	GSE3311	*Rattus norvegicus*	Alcohol poisoning	Pancreas
333	0.51	6.03E-01	7.21E-01	GSE2503	*Homo sapiens*	Squamous cell carcinoma	Skin tissue
334	0.5	6.17E-01	7.35E-01	GSE2899	*Mus musculus*	Type 2 diabetes mellitus	Hepatic tissue
335	0.48	6.26E-01	7.42E-01	GSE1739	*Homo sapiens*	SARS	Peripheral blood mononuclear cell
336	0.46	6.46E-01	7.57E-01	GSE5900	*Homo sapiens*	Smoldering multiple myeloma	Bone marrow
337	0.45	6.64E-01	7.74E-01	GSE4678	*Mus musculus*	Ventricular hypertrophy	Myocardial tissue
338	0.43	6.63E-01	7.74E-01	GSE4128	*Mus musculus*	Adenovirus infection	Hepatic tissue
339	0.42	6.79E-01	7.81E-01	GSE11889	*Homo sapiens*	CML	Hematopoietic stem cell
340	0.42	6.72E-01	7.79E-01	GSE2779	*Homo sapiens*	Vitamin B 12 deficiency	Bone marrow stem cell
341	0.4	6.89E-01	7.90E-01	GSE3	*Homo sapiens*	Clear cell carcinoma of kidney	Renal cell
342	0.38	7.02E-01	7.97E-01	GSE9692	*Homo sapiens*	Septic shock	Whole blood
343	0.38	7.10E-01	8.01E-01	GSE6740	*Homo sapiens*	HIV	T lymphocyte
344	0.36	7.13E-01	8.03E-01	GSE121	*Homo sapiens*	Type 2 diabetes mellitus	Muscle tissue
345	0.36	7.25E-01	8.14E-01	GSE1402	*Homo sapiens*	Spondyloarthropathy	Peripheral blood mononuclear cell
346	0.34	7.34E-01	8.20E-01	GSE2271	*Mus musculus*	Hypoxia	Heart
347	0.34	7.40E-01	8.21E-01	GSE2466	*Homo sapiens*	Lymphocytic leukemia, chronic, B Cell	PBL
348	0.34	7.37E-01	8.21E-01	GSE1751	*Homo sapiens*	Huntington's disease	Blood
349	0.28	7.76E-01	8.48E-01	GSE10211	*Mus musculus*	Sendai virus infection	Tracheal epithelium
350	0.27	7.86E-01	8.57E-01	GSE2379	*Homo sapiens*	Hypopharyngeal cancer	Pharynx
351	0.25	8.01E-01	8.70E-01	GSE2884	*Rattus norvegicus*	Neurological pain disorder	Dorsal root ganglia
352	0.24	8.02E-01	8.70E-01	GSE1560	*Mus musculus*	Atherosclerosis	Aorta smooth muscle tissue
353	0.19	8.51E-01	9.11E-01	GSE18803	*Rattus norvegicus*	Neurological pain disorder	CNS, spinal cord (MMHCC)
354	0.17	8.63E-01	9.18E-01	GSE1124	*Homo sapiens*	Malaria	Blood
355	0.17	8.69E-01	9.19E-01	EXPE-MEXP-1050	*Homo sapiens*	IUGR	Decidua basalis
356	0.15	8.82E-01	9.28E-01	GSE4697	*Mus musculus*	Obesity	Adipose tissue
357	0.12	9.02E-01	9.43E-01	GSE3249	*Mus musculus*	Leber congenital amaurosis	Retina
358	0.12	9.02E-01	9.43E-01	GSE1852	*Mus musculus*	Marfan’s syndrome	Myocardial tissue
359	0.08	9.34E-01	9.66E-01	GSE7654	*Rattus norvegicus*	Hepatitis	Liver
360	0.08	9.35E-01	9.66E-01	GSE2504	*Homo sapiens*	HIV	T lymphocyte
361	0.08	9.39E-01	9.66E-01	GSE1710	*Homo sapiens*	Ulcerative colitis	Sigmoid colon
362	0.08	9.40E-01	9.66E-01	GSE3167	*Homo sapiens*	Urothelial carcinoma	Urothelium
363	0.07	9.45E-01	9.66E-01	GSE5281	*Homo sapiens*	Alzheimer's disease	Entorhinal cortex
364	0.07	9.46E-01	9.66E-01	GSE3586	*Homo sapiens*	Cardiomyopathy, dilated	Myocardial tissue
365	0.04	9.70E-01	9.88E-01	GSE10758	*Homo sapiens*	Down syndrome	Fetus
366	0.02	9.82E-01	9.89E-01	GSE15966	*Homo sapiens*	Gastrointestinal stromal tumor	Gastric tissue
367	0.01	9.94E-01	9.96E-01	GSE11969	*Homo sapiens*	Small cell carcinoma of lung	Lung tissue
368	0	9.98E-01	9.98E-01	GSE4482	*Homo sapiens*	Cancer of cervix	Cervix
369	−0.01	9.93E-01	9.96E-01	GSE11393	*Homo sapiens*	Familial combined hyperlipidemia	Blood monocyte
370	−0.02	9.81E-01	9.89E-01	GSE10474	*Homo sapiens*	Acute lung injury	Whole blood
371	−0.02	9.81E-01	9.89E-01	GSE574	*Homo sapiens*	Purpura, idiopathic thrombocytopenic	T lymphocyte
372	−0.02	9.80E-01	9.89E-01	GSE3827	*Homo sapiens*	CBCL	Skin tissue
373	−0.03	9.76E-01	9.89E-01	GSE8835	*Homo sapiens*	B-cell chronic lymphocytic leukemia/small lymphocytic lymphoma	Peripheral blood mononuclear cell
374	−0.07	9.42E-01	9.66E-01	GSE2779	*Homo sapiens*	Folic acid deficiency	Bone marrow stem cell
375	−0.1	9.19E-01	9.54E-01	GSE860	*Homo sapiens*	PTSD	Peripheral blood mononuclear cell
376	−0.11	9.18E-01	9.54E-01	GSE2935	*Mus musculus*	Sendai virus infection	Macrophage
377	−0.11	9.15E-01	9.54E-01	GSE4483	*Homo sapiens*	Hypoxia	Astrocyte
378	−0.14	8.89E-01	9.34E-01	GSE1650	*Homo sapiens*	COPD	Lung tissue
379	−0.16	8.74E-01	9.23E-01	EXPE-MEXP-1278	*Rattus norvegicus*	Arteriotomy	Carotid artery
380	−0.17	8.68E-01	9.19E-01	GSE1541	*Homo sapiens*	Lung injury	Lung tissue
381	−0.18	8.52E-01	9.11E-01	GSE2899	*Mus musculus*	Type 2 diabetes mellitus	pancreatic islet
382	−0.19	8.55E-01	9.12E-01	GSE935	*Homo sapiens*	Granulomatous disease, chronic	Blood neutrophil
383	−0.19	8.42E-01	9.06E-01	GSE2171	*Homo sapiens*	HIV infection	Peripheral blood mononuclear cell
384	−0.2	8.46E-01	9.08E-01	GSE4036	*Homo sapiens*	Schizophrenia	CNS, brain, cerebellum (MMHCC)
385	−0.22	8.27E-01	8.93E-01	GSE14245	*Homo sapiens*	Malignant tumor of pancreas	Saliva
386	−0.24	8.06E-01	8.72E-01	GSE2459	*Mus musculus*	Hypertrophy, left ventricular	Myocardial tissue
387	−0.29	7.69E-01	8.44E-01	GSE1818	*Homo sapiens*	Cancer of the testis	Testis
388	−0.29	7.71E-01	8.44E-01	GSE10789	*Homo sapiens*	Leukemia, adult T cell	Blood monocyte
389	−0.3	7.66E-01	8.43E-01	GSE2368	*Mus musculus*	Ventilator-associated lung injury	Lung tissue
390	−0.32	7.59E-01	8.38E-01	GSE11	*Mus musculus*	Type 1 diabetes mellitus	Splenic tissue
391	−0.32	7.44E-01	8.24E-01	GSE2507	*Mus musculus*	Muscular dystrophy	Myocardial tissue
392	−0.34	7.39E-01	8.21E-01	GSE11969	*Homo sapiens*	Adenocarcinoma of lung	Lung tissue
393	−0.34	7.29E-01	8.17E-01	GSE3	*Homo sapiens*	Chromophil carcinoma of kidney	Renal cell
394	−0.37	7.05E-01	7.99E-01	GSE1710	*Homo sapiens*	Crohn's disease	Sigmoid colon
395	−0.38	6.97E-01	7.93E-01	GSE1987	*Homo sapiens*	Squamous cell carcinoma	Lung tissue
396	−0.4	6.96E-01	7.93E-01	GSE1719	*Homo sapiens*	Macular degeneration	Fibroblast
397	−0.4	6.91E-01	7.90E-01	GSE2737	*Homo sapiens*	Psoriasis vulgaris	Epidermis
398	−0.41	6.78E-01	7.81E-01	GSE2729	*Homo sapiens*	Rotavirus infection of children	Peripheral blood mononuclear cell
399	−0.41	6.78E-01	7.81E-01	GSE14577	*Homo sapiens*	CFS	Peripheral blood mononuclear cell
400	−0.43	6.66E-01	7.74E-01	GSE363	*Mus musculus*	Atherosclerosis	Hepatic tissue
401	−0.46	6.46E-01	7.57E-01	GSE1987	*Homo sapiens*	Adenocarcinoma of lung	Lung tissue
402	−0.47	6.42E-01	7.57E-01	GSE1786	*Homo sapiens*	Senescence	Muscle, striated (skeletal) (MMHCC)
403	−0.47	6.33E-01	7.48E-01	GSE2018	*Homo sapiens*	Lung transplant rejection	Lung tissue
404	−0.49	6.26E-01	7.42E-01	GSE5113	*Mus musculus*	Intestinal flagellate infection	Small intestinal lamina propria
405	−0.52	5.99E-01	7.19E-01	GSE4646	*Homo sapiens*	Meningococcal infection	Umbilical vein
406	−0.54	5.89E-01	7.15E-01	GSE3934	*Homo sapiens*	Meningococcal infection	Blood
407	−0.54	5.86E-01	7.13E-01	GSE11969	*Homo sapiens*	Squamous cell carcinoma of lung	Lung tissue
408	−0.55	5.80E-01	7.08E-01	GSE3606	*Homo sapiens*	Overexertion	Leukocyte
409	−0.59	5.55E-01	6.99E-01	GSE2378	*Homo sapiens*	Glaucoma	Astrocyte
410	−0.61	5.43E-01	6.86E-01	GSE11969	*Homo sapiens*	Large cell carcinoma of lung	Lung tissue
411	−0.63	5.34E-01	6.77E-01	GSE1907	*Homo sapiens*	Sarcoidosis	Blood corpuscle
412	−0.7	4.74E-01	6.17E-01	GSE3100	*Mus musculus*	Cystic fibrosis	Lung
413	−0.74	4.62E-01	6.07E-01	GSE7277	*Mus musculus*	Arthritis	frontal cortex
414	−0.85	3.97E-01	5.39E-01	GSE3384	*Mus musculus*	Nemaline myopathy	Gastrocnemius muscle
415	−0.85	3.95E-01	5.38E-01	GSE1363	*Mus musculus*	Primary pulmonary hypoplasia	Lung tissue
416	−0.86	3.85E-01	5.26E-01	GSE11886	*Homo sapiens*	Ankylosing spondylitis	macrophage
417	−0.9	3.66E-01	5.05E-01	GSE1542	*Homo sapiens*	Duct cell carcinoma	Pancreas
418	−0.94	3.40E-01	4.81E-01	GSE3414	*Mus musculus*	SCID	Lung tissue
419	−1.04	2.98E-01	4.35E-01	GSE2223	*Homo sapiens*	Anaplastic oligoastrocytoma	CNS, brain (MMHCC)
420	−1.11	2.66E-01	3.97E-01	GSE1615	*Homo sapiens*	Polycystic ovary syndrome	Theca cells
421	−1.17	2.41E-01	3.70E-01	GSE1402	*Homo sapiens*	Chronic polyarticular juvenile rheumatoid arthritis	Peripheral blood mononuclear cell
422	−1.18	2.37E-01	3.65E-01	GSE5786	*Mus musculus*	Huntington's disease	CNS, brain, striatum (MMHCC)
423	−1.23	2.19E-01	3.42E-01	GSE3284	*Homo sapiens*	Bacterial infection	Leukocyte
424	−1.28	2.01E-01	3.18E-01	GSE3512	*Rattus norvegicus*	Hyperlipidemia	Hepatic tissue
425	−1.29	1.97E-01	3.16E-01	EXPE-MEXP-357	*Rattus norvegicus*	Hypertension	Left ventricle
426	−1.36	1.75E-01	2.88E-01	GSE4630	*Homo sapiens*	Hypoxia	macrophage
427	−1.38	1.68E-01	2.80E-01	GSE1294	*Mus musculus*	Down’s syndrome	CNS, brain (MMHCC)
428	−1.39	1.61E-01	2.70E-01	GSE5112	*Mus musculus*	Intestinal flagellate infection	Intestinal epithelium
429	−1.42	1.53E-01	2.61E-01	GSE2006	*Homo sapiens*	Essential thrombocythemia	Thrombocyte
430	−1.44	1.46E-01	2.52E-01	GSE1402	*Homo sapiens*	Pauciarticular juvenile arthritis	Peripheral blood mononuclear cell
431	−1.54	1.24E-01	2.25E-01	EXPE-MEXP-1283	*Homo sapiens*	Hodgkin’s lymphoma	Peripheral blood mononuclear cell
432	−1.63	1.03E-01	1.94E-01	GSE1919	*Homo sapiens*	RA	Synovial membrane
433	−1.73	8.56E-02	1.69E-01	GSE2395	*Homo sapiens*	Cystic fibrosis	Bronchial epithelium
434	−1.74	8.09E-02	1.60E-01	GSE6435	*Mus musculus*	Bacterial infection	Leukocyte, monocyte, macrophage (MMHCC)
435	−1.76	7.44E-02	1.50E-01	GSE1919	*Homo sapiens*	Osteoarthritis	Synovial membrane
436	−2.77	4.80E-03	1.74E-02	GSE10121	*Homo sapiens*	Squamous cell carcinoma of buccal mucosa	Buccal mucosa

We computationally matched the juvenile plasticity signature to 436 disease signatures derived from public microarray data. This systematic method applies a rank-based molecular matching algorithm to determine the molecular concordance between the plasticity signature and a given disease signature, where high scores indicate that plasticity genes are significantly dysregulated by the disease and low scores indicate that the disease has no impact on plasticity genes. Highly ranked diseases included not only brain disorders known to disrupt plasticity such as Huntington’s disease, but also non-neurologic disorders (e.g., bacterial infections, inflammatory bowel disease, metabolic diseases), suggesting that a broad range of disease states may impact molecular pathways involved in plasticity. ALS, Amyotrophic lateral sclerosis; AML, acute myeloid leukemia; APECED, autoimmune polyendocrinopathy-candidiasis-ectodermal dystrophy; CBCL, cutaneous B-cell lymphoma; CFS, chronic fatigue syndrome; CML, chronic myeloid leukemia; COPD, chronic obstructive pulmonary disease; DMD, Duchenne muscular dystrophy; FDIU, fetal death in utero; FDR, false discovery rate; GERD, gastroesophageal reflux disease; HIV, human immunodeficiency virus; IBD, inflammatory bowel disease; IBS, irritable bowel syndrome; IUGR, intrauterine growth retardation; JRA, juvenile rheumatoid arthritis; LGLL, large granular lymphocytic leukemia; MCTD, mixed connective tissue disease; MDS, myelodysplastic syndrome; MGUS, Monoclonal gammopathy of undetermined significance; MMHCC, Mouse Models of Human Cancers Consortium; MODY, maturity onset diabetes in youth type 1; MS, multiple sclerosis; PBL, peripheral blood lymphocyte; PTSD, post-traumatic stress disorder; RA, rheumatoid arthritis; SARS, severe acute respiratory syndrome; SCID, severe combined immunodeficiency.

Finally, we tested whether inflammation suppresses experience-dependent developmental cortical plasticity *in vivo*. We administered LPS (300 μg/kg, intraperitoneal) or saline on P26 immediately after suturing one eye to induce ocular dominance plasticity via monocular deprivation (MD; Fig. 4a). After 3 d of MD, we conducted *in vivo* single-unit recordings of activity-driven changes in the eye preference of single neurons (ocular dominance) in binocular V1 in response to light (Gordon and Stryker, 1996). In mice treated with saline, we observed the expected shift in cortical responsivity to light stimulation from the deprived contralateral to nondeprived ipsilateral eye, as quantified by a decrease in the animal-level CBI (CBI = 0.49 ± 0.04; six mice; 135 cells), indicating the presence of developmental plasticity (Fig. 4c, right-hand plot). In contrast, LPS significantly suppressed the shift in cortical responsivity from the deprived contralateral eye to the nondeprived ipsilateral eye, which was quantified by an increase in CBI and an elimination of the right shift in the distribution of ODSs of single neurons (CBI = 0.64 ± 0.02, seven mice, 129 cells; one-sided *t* test of CBIs: *p* = 0.006_l_; χ^2^ test of ODS distribution: *p* = 2.6 × 10^−6^_m_), indicating impaired plasticity during the critical period in V1 (Fig. 4c, left-hand plot). Together, these data are consistent with our informatics-derived hypothesis by demonstrating that peripheral injection of LPS induces an inflammatory response in the brain and suppresses developmental cortical plasticity *in vivo.*

## Discussion

Using an integrative bioinformatics approach, we found that inflammation disrupts developmental cortical plasticity. Our study demonstrates the utility of this approach for both identifying diseases that may disrupt plasticity and generating hypotheses on the molecular mechanisms underlying these disruptions. Moreover, our novel Disease Leverage Analysis facilitates novel hypothesis generation, because seemingly unrelated phenotypes, such as neuroplasticity and inflammation, can be connected based on apparently disparate tissues and diseases. Previous work indicating that the disease signal harmonizes across tissues (Dudley et al., 2009) supports this approach and in the present study suggests that the molecular pathways underlying plasticity are shared in diverse tissues and are dysregulated in many disease states, including apparently non-neurological phenotypes (e.g., bacterial infections, inflammatory bowel disease, metabolic diseases). Importantly, the biological relevance of any given molecular match between plasticity and disease must be interpreted with care. In all cases, molecular matches indicate that plasticity and the disease phenotype share underlying molecular machinery. However, a given disease state in a specific tissue may or may not have an impact on functional plasticity or related gene expression if the disease state or tissue is sufficiently localized and segregated from neural tissue. Consequently, we developed Disease Leverage Analysis to use the information of all matches collectively to identify common disease processes and simultaneously shrink the hypothesis space to a manageable set of disease process-oriented hypotheses that bind together the diverse matches. This approach facilitated the unbiased and systematic use of apparently disparate disease signatures to generate novel hypotheses about shared disease mechanisms that may dysregulate plasticity. We find here that a common theme among these dysregulations is inflammation, a biological process that is well suited to communicate peripheral signals to the brain, disrupting plasticity.

We demonstrate several lines of evidence supporting a hypothesis that plasticity and inflammatory processes share components of underlying molecular networks. We computationally predicted associations between plasticity signature-perturbing diseases and TNF-α and IFN-γ pathways (Figs. 1, 2) and also experimentally identified associations between systemic inflammation and increases in the plasticity brakes *PirB* and *MHC-1* in the brain (Fig. 4). These predictions and observations confirm the known role of pathways involving TNF-α, IFN-γ, Pirb, and MHC-I on regulating developmental plasticity (Syken et al., 2006; Kaneko et al., 2008; Datwani et al., 2009; Nagakura et al., 2014) and extend them to the context of inflammation. We also showed that *BDNF*, a neurotrophic factor essential to the opening of the critical period (Huang et al., 1999), is decreased after LPS administration (Fig. 4), which is consistent with the reported antagonistic relationship of peripheral LPS with BDNF (Guan and Fang, 2006; Schnydrig et al., 2007). Interestingly, we also found that the microglial activator Lcn2 (Jang et al., 2013) is a member of both the juvenile and *Lynx1*^−/−^ plasticity signatures (Tables 2, 4) and is dramatically increased after LPS in V1 during the critical period (Fig. 3). Activation may inhibit microglia from carrying out their “resting-state” role in mediating experience-dependent plasticity (Sipe et al., 2016), contributing to the dampening of plasticity by inflammation. Collectively, our work suggests a conflict between developmental cortical plasticity and immune-related molecular networks during inflammation, ultimately resulting in the suppression of plasticity during inflammation. Our study provides a novel subset of transcripts that can be used to guide future mechanistic studies into inflammation–plasticity interactions.

**Table 4:** *Lynx1*^−/−^ plasticity signature

RP	FC	pfp	*p* value	Probe_id	Symbol	mm_Entrez_ID	hs_Entrez_ID	Gene_name
1.2599	6.1102	0	0	ILMN_1213274	Ydjc	69101	150223	YdjC homolog (bacterial)
26.1378	1.722	0	0	ILMN_2744660	Igh-6	NA	NA	NA
87.0435	1.5896	0.0325	0	ILMN_1259747	Il33	77125	90865	Interleukin-33
79.5578	1.583	0.0233	0	ILMN_1231445	Inmt	21743	11185	Indolethylamine *N*-methyltransferase
99.1693	1.5584	0.0189	0	ILMN_1252675	D230019G01Rik	NA	NA	NA
95.5604	1.5581	0.0267	0	ILMN_2499166	Tmem65	74868	157378	Transmembrane protein 65
98.106	1.5481	0.0213	0	ILMN_1251713	Car12	76459	771	Carbonic anyhydrase 12
94.6178	1.5436	0.032	0	ILMN_1244887	Gria4	NA	NA	NA
96.4906	1.5339	0.0229	0	ILMN_2649671	Ipas	53417	64344	Hypoxia-inducible factor 3, α subunit
122.0152	1.5276	0.035	0	ILMN_2680188	Bai1	107831	575	Brain-specific angiogenesis inhibitor 1
137.2649	1.5013	0.04	0	ILMN_2968369	Kcnip1	70357	30820	Kv channel-interacting protein 1
128.559	1.4989	0.0418	0	ILMN_2744657	Igh-6	NA	NA	NA
135.6589	1.4911	0.0425	0	ILMN_3049896	Evi2b	NA	NA	NA
295.2115	−1.4885	0.0464	3.00E-04	ILMN_2847144	Hist1h2ak	319169	221613	Histone cluster 1, H2ak
271.4427	−1.5015	0.0389	2.00E-04	ILMN_2619455	Mcat	223722	27349	Malonyl CoA:ACP acyltransferase (mitochondrial)
309.8626	−1.5083	0.0494	3.00E-04	ILMN_2700265	Pex11a	18631	8800	Peroxisomal biogenesis factor 11 alpha
310.3477	−1.5135	0.0487	3.00E-04	ILMN_1254828	A730014O07Rik	NA	NA	NA
306.798	−1.5145	0.049	3.00E-04	ILMN_2684693	Dusp3	72349	1845	Dual-specificity phosphatase 3 (vaccinia virus phosphatase VH1-related)
307.0792	−1.5179	0.0488	3.00E-04	ILMN_2869715	Nell2	54003	4753	NEL-like 2
277.2855	−1.5191	0.0393	2.00E-04	ILMN_2491589	Vgll4	232334	9686	Vestigial like 4 (Drosophila)
311.6705	−1.5218	0.0487	4.00E-04	ILMN_2568488	A530089A20Rik	218914	23063	Wings apart-like homolog (Drosophila)
300.5669	−1.5221	0.0474	3.00E-04	ILMN_2768841	Rab22a	NA	NA	NA
312.3161	−1.5221	0.0484	4.00E-04	ILMN_3163448	Cdh8	12564	1006	Cadherin 8
261.4704	−1.5237	0.0365	2.00E-04	ILMN_1214071	Ifitm1	68713	NA	Interferon-induced transmembrane protein 1
273.6424	−1.5279	0.0394	2.00E-04	ILMN_2757232	Aqp4	11829	361	aquaporin 4
285.8745	−1.5314	0.0422	3.00E-04	ILMN_1241551	Nell2	54003	4753	NEL-like 2
240.2307	−1.534	0.0322	2.00E-04	ILMN_2629112	Asah3l	230379	340485	Alkaline ceramidase 2
295.481	−1.5354	0.046	3.00E-04	ILMN_2688728	Pcyt1a	13026	5130	Phosphate cytidylyltransferase 1, choline, α isoform
249.2494	−1.5366	0.0328	2.00E-04	ILMN_3151149	Cpne8	66871	144402	Copine VIII
273.9191	−1.5385	0.0391	2.00E-04	ILMN_2657911	Cnot4	53621	4850	CCR4-NOT transcription complex, subunit 4
309.8885	−1.5389	0.049	3.00E-04	ILMN_1257190	2900083I11Rik	58212	NA	Serine/arginine repetitive matrix 3
269.2151	−1.5392	0.0385	2.00E-04	ILMN_2639849	Cbln4	228942	140689	Cerebellin 4 precursor protein
290.2289	−1.5404	0.0445	3.00E-04	ILMN_1255457	Crebbp	12914	1387	CREB binding protein
256.2379	−1.5408	0.0356	2.00E-04	ILMN_3072536	Eif5	217869	1983	Eukaryotic translation initiation factor 5
242.1891	−1.542	0.0315	2.00E-04	ILMN_1249000	1500015O10Rik	78896	84417	RIKEN cDNA 1500015O10 gene
306.5435	−1.5432	0.0494	3.00E-04	ILMN_1214405	Cnksr2	245684	22866	Connector enhancer of kinase suppressor of Ras 2
278.6573	−1.5516	0.0393	2.00E-04	ILMN_2751988	Kitl	17311	4254	Kit ligand
274.1734	−1.5518	0.0391	2.00E-04	ILMN_2673566	Eif4ebp2	13688	1979	Eukaryotic translation initiation factor 4E binding protein 2
274.7663	−1.5538	0.0391	2.00E-04	ILMN_1216085	B230387C07Rik	106585	23253	Ankyrin repeat domain 12
267.3929	−1.5547	0.0379	2.00E-04	ILMN_2703913	Mtf2	17765	22823	Metal response element binding transcription factor 2
218.0454	−1.5569	0.03	1.00E-04	ILMN_2535881	Macrod2	72899	140733	MACRO domain containing 2
298.574	−1.5571	0.0468	3.00E-04	ILMN_1246494	LOC381445	26422	26960	Neurobeachin
240.5714	−1.5576	0.0318	2.00E-04	ILMN_1225224	Ttc14	67120	151613	Tetratricopeptide repeat domain 14
257.4399	−1.5601	0.0364	2.00E-04	ILMN_1259724	Man1b	17156	10905	Mannosidase, α, class 1A, member 2
225.5851	−1.5603	0.0305	1.00E-04	ILMN_1227376	BC042720	329178	285175	unc-80 homolog (*Caenorhabditis elegans*)
233.0899	−1.5645	0.0306	1.00E-04	ILMN_2741677	Bach1	12013	571	BTB and CNC homology 1
241.988	−1.5645	0.0319	2.00E-04	ILMN_1230312	A830054O04Rik	NA	NA	NA
309.3927	−1.5684	0.0497	3.00E-04	ILMN_2793638	H2-Tw3	NA	NA	NA
275.3065	−1.5696	0.0391	2.00E-04	ILMN_2757889	Zfp91-cntf	NA	NA	NA
225.8264	−1.5706	0.0301	1.00E-04	ILMN_2654822	Chd4	107932	1108	Chromodomain helicase DNA binding protein 4
247.466	−1.5728	0.0324	2.00E-04	ILMN_2731854	Creg2	263764	200407	Cellular repressor of E1A-stimulated genes 2
218.4269	−1.5755	0.0297	1.00E-04	ILMN_1221598	9330133O14Rik	NA	NA	NA
259.2859	−1.578	0.0363	2.00E-04	ILMN_1227149	Meg3	17263	NA	Maternally expressed 3
245.0182	−1.579	0.032	2.00E-04	ILMN_2601176	Meg3	17263	NA	Maternally expressed 3
246.2646	−1.5793	0.0323	2.00E-04	ILMN_1230605	Gm336	212285	116984	ArfGAP with RhoGAP domain, ankyrin repeat and PH domain 2
225.2295	−1.5805	0.0309	1.00E-04	ILMN_2638066	Gria3	53623	2892	Glutamate receptor, ionotropic, AMPA3 (alpha 3)
224.7568	−1.5808	0.0311	1.00E-04	ILMN_1253224	Dhcr24	74754	1718	24-Dehydrocholesterol reductase
204.3878	−1.5825	0.0256	1.00E-04	ILMN_2544603	2610015J01Rik	67039	NA	RNA binding motif protein 25
190.3687	−1.5853	0.0233	1.00E-04	ILMN_2593230	Mllt4	17356	4301	Myeloid/lymphoid or mixed-lineage leukemia (trithorax homolog, Drosophila); translocated to, 4
190.5304	−1.5853	0.0225	1.00E-04	ILMN_2519673	Vwf	22371	7450	Von Willebrand factor homolog
194.1056	−1.5868	0.0229	1.00E-04	ILMN_1215713	Egr4	13656	1961	Early growth response 4
220.9951	−1.5896	0.0293	1.00E-04	ILMN_1239599	Bat2d	226562	23215	Proline-rich coiled-coil 2C
218.0063	−1.5911	0.0304	1.00E-04	ILMN_2637413	Dbpht2	386753	NA	DNA binding protein with his-thr domain
185.7923	−1.5957	0.023	1.00E-04	ILMN_1247295	Hook1	77963	51361	Hook homolog 1 (Drosophila)
225.437	−1.5957	0.0309	1.00E-04	ILMN_2520582	Plxna4	243743	91584	Plexin A4
204.4627	−1.6008	0.0249	1.00E-04	ILMN_2674890	Tbl1x	21372	6907	Transducin (β)-like 1 X-linked
198.2794	−1.6018	0.0238	1.00E-04	ILMN_2541675	LOC382128	319675	85459	RIKEN cDNA 5830418K08 gene
202.278	−1.6049	0.0251	1.00E-04	ILMN_2690603	Spp1	20750	6696	Secreted phosphoprotein 1
204.4135	−1.6064	0.0252	1.00E-04	ILMN_1224992	Gcap14	72972	54462	Coiled-coil serine rich 2
236.1982	−1.609	0.0309	2.00E-04	ILMN_1235124	Thsd4	207596	79875	Thrombospondin, type I, domain containing 4
193.5829	−1.616	0.0231	1.00E-04	ILMN_2737867	Mtap1b	17755	4131	Microtubule-associated protein 1B
219.8212	−1.616	0.0296	1.00E-04	ILMN_1254547	Nr4a2	18227	4929	Nuclear receptor subfamily 4, group A, member 2
174.6364	−1.6168	0.0212	1.00E-04	ILMN_1235652	Usp37	319651	57695	Ubiquitin-specific peptidase 37
190.4584	−1.6202	0.0229	1.00E-04	ILMN_1255854	Mtap9	213582	79884	Microtubule-associated protein 9
183.2527	−1.6215	0.0226	1.00E-04	ILMN_3114632	Ddx6	13209	1656	DEAD (Asp-Glu-Ala-Asp) box polypeptide 6
168.6428	−1.6221	0.0204	1.00E-04	ILMN_2561533	Vps13a	271564	23230	Vacuolar protein sorting 13A (yeast)
169.7224	−1.6236	0.0204	1.00E-04	ILMN_1241229	Bat2d	226562	23215	Proline-rich coiled-coil 2C
161.0071	−1.6316	0.0176	0	ILMN_1231734	Nsd1	18193	64324	Nuclear receptor-binding SET-domain protein 1
228.5556	−1.6327	0.0303	1.00E-04	ILMN_1259781	A430041B07Rik	328108	23116	Family with sequence similarity 179, member B
164.2141	−1.6332	0.0193	1.00E-04	ILMN_1250030	Cdc42ep1	104445	11135	CDC42 effector protein (Rho GTPase binding) 1
186.6603	−1.6332	0.0229	1.00E-04	ILMN_2795040	Hist1h2ad	NA	NA	NA
189.467	−1.6364	0.0239	1.00E-04	ILMN_1216322	Hmgcs2	15360	3158	3-Hydroxy-3-methylglutaryl-coenzyme A synthase 2
189.7896	−1.6367	0.0235	1.00E-04	ILMN_2646070	Mtap1a	17754	4130	Microtubule-associated protein 1 A
173.3318	−1.6391	0.0212	1.00E-04	ILMN_1229727	Gpr123	52389	84435	G-protein-coupled receptor 123
182.5954	−1.642	0.0227	1.00E-04	ILMN_2715848	Slitrk4	245446	139065	SLIT and NTRK-like family, member 4
154.7165	−1.6445	0.0157	0	ILMN_2481391	Zfp326	54367	284695	Zinc finger protein 326
144.623	−1.6461	0.0132	0	ILMN_1224129	Dennd3	105841	22898	DENN/MADD domain containing 3
175.2067	−1.6461	0.0208	1.00E-04	ILMN_2445958	Tssc8	63830	NA	KCNQ1 overlapping transcript 1
201.9407	−1.6496	0.0253	1.00E-04	ILMN_3059326	Sparc	20692	6678	Secreted acidic cysteine-rich glycoprotein
153.5956	−1.6636	0.0154	0	ILMN_3031781	Arid5b	71371	84159	AT-rich interactive domain 5B (MRF1-like)
140.1691	−1.6694	0.0126	0	ILMN_2481389	Zfp326	54367	284695	Zinc finger protein 326
134.6324	−1.67	0.0109	0	ILMN_1257525	Cpeb3	208922	22849	Cytoplasmic polyadenylation element binding protein 3
135.784	−1.6706	0.0108	0	ILMN_1237548	5830407P18Rik	NA	NA	NA
158.0665	−1.6714	0.0161	0	ILMN_1220048	A330086O21Rik	434089	NA	Predicted gene 10010
131.7096	−1.672	0.0116	0	ILMN_1254296	Chd1	12648	1105	Chromodomain helicase DNA binding protein 1
134.8336	−1.6821	0.0106	0	ILMN_2654932	Pdap1	231887	11333	PDGFA-associated protein 1
156.3313	−1.6827	0.016	0	ILMN_2457731	A930010C08Rik	NA	NA	NA
124.0646	−1.6846	0.0117	0	ILMN_2747381	Ddx24	27225	57062	DEAD (Asp-Glu-Ala-Asp) box polypeptide 24
138.2192	−1.6875	0.0114	0	ILMN_1248181	Zbtb7a	16969	51341	Zinc finger and BTB domain containing 7a
109.4147	−1.7232	0.0089	0	ILMN_2492395	2900064A13Rik	NA	NA	NA
127.2584	−1.7307	0.0116	0	ILMN_1251488	A430041B07Rik	328108	23116	Family with sequence similarity 179, member B
93.3629	−1.7373	0.0064	0	ILMN_1218471	3-Sep	24050	55964	Septin 3
93.4053	−1.747	0.0062	0	ILMN_2675914	R3hdm1	226412	23518	R3H domain containing 1
124.2134	−1.7587	0.0113	0	ILMN_2680128	Zc3h13	67302	NA	Zinc finger CCCH type containing 13
80.5466	−1.7652	0.0055	0	ILMN_1239042	Ankhd1	108857	54882	Ankyrin repeat and KH domain containing 1
86.1003	−1.7765	0.0054	0	ILMN_3162820	Odz4	23966	26011	Teneurin transmembrane protein 4
82.5619	−1.7931	0.0057	0	ILMN_1236820	9430047F21Rik	NA	NA	NA
134.4843	−1.794	0.0112	0	ILMN_2750515	Fos	14281	2353	FBJ osteosarcoma oncogene
71.9896	−1.8054	0.0055	0	ILMN_1258834	Chd1	12648	1105	Chromodomain helicase DNA binding protein 1
141.8688	−1.8109	0.0128	0	ILMN_3035795	Mllt3	70122	4300	Myeloid/lymphoid or mixed-lineage leukemia (trithorax homolog, Drosophila); translocated to, 3
271.3241	−1.8126	0.0392	2.00E-04	ILMN_2965669	Xlr4a	NA	NA	NA
58.7952	−1.8477	0.0031	0	ILMN_2589525	Cpeb3	208922	22849	Cytoplasmic polyadenylation element binding protein 3
108.4816	−1.8546	0.0093	0	ILMN_2803674	S100a9	20202	6280	S100 calcium-binding protein A9 (calgranulin B)
73.6082	−1.8653	0.0057	0	ILMN_2639442	Rock2	19878	9475	Rho-associated coiled-coil containing protein kinase 2
65.4886	−1.8737	0.0032	0	ILMN_2617920	1110017D15Rik	73721	84688	RIKEN cDNA 1110017D15 gene
48.1193	−1.9436	0.0021	0	ILMN_1217776	Mll5	69188	55904	Lysine (K)-specific methyltransferase 2E
53.5131	−1.9814	0.0027	0	ILMN_2702303	Ch25h	12642	9023	Cholesterol 25-hydroxylase
43.2714	−2.0064	0.0015	0	ILMN_2649773	Slc38a5	209837	92745	Solute carrier family 38, member 5
37.5276	−2.0092	9.00E-04	0	ILMN_1244343	B230369L08Rik	223697	25777	Sad1 and UNC84 domain containing 2
41.0522	−2.0325	0.0017	0	ILMN_1226085	Syt1	NA	NA	NA
30.3722	−2.0661	0.0011	0	ILMN_1256701	2900016B01Rik	74901	9920	Kelch repeat and BTB (POZ) domain containing 11
33.181	−2.0665	0.001	0	ILMN_2778076	2210021J22Rik	72355	150383	Cysteine-rich, DPF motif domain containing 1
61.2262	−2.0942	0.0029	0	ILMN_2668333	Prg4	96875	NA	Proteoglycan 4 (megakaryocyte stimulating factor, articular superficial zone protein)
61.9241	−2.1057	0.0033	0	ILMN_2623983	Egr2	13654	1959	Early growth response 2
18.8649	−2.4826	0.0014	0	ILMN_2652500	Lrg1	76905	116844	Leucine-rich alpha-2-glycoprotein 1
13.3721	−2.4851	0	0	ILMN_2654906	Mgat3	17309	4248	Mannoside acetylglucosaminyltransferase 3
25.6626	−2.6212	0.0012	0	ILMN_2710905	S100a8	20201	6279	S100 calcium binding protein A8 (calgranulin A)
6.5864	−3.7552	0	0	ILMN_2712075	Lcn2	16819	3934	Lipocalin 2
4.4996	−4.7574	0	0	ILMN_1243615	Gramd4	223752	23151	GRAM domain containing 4
3.677	−5.3591	0	0	ILMN_1223734	Atf4	11911	468	Activating transcription factor 4
2.1886	−7.3314	0	0	ILMN_2507182	Tomm22	223696	56993	Translocase of outer mitochondrial membrane 22 homolog (yeast)
1	−13.9276	0	0	ILMN_1256369	Lynx1	23936	66004	Ly6/neurotoxin 1

V1 transcriptomes were profiled with microarray from *Lynx1*^−/−^ mice (the plasticity brake Lynx1 is genetically deleted to allow experience-dependent plasticity even in adulthood) to generate the *Lynx1*^−/−^ plasticity differential expression signature. Using RankProd differential expression (DE) analysis, V1 of adult *Lynx1*^−/−^ male mice >P60 was compared with adult wild-type mice (> P60; *n* = 3 each group) to identify 132 DE probes, which mapped to 107 unique mouse Entrez IDs. For downstream analysis, mouse Entrez IDs were mapped to human orthologs using the Mouse Genome Informatics homology reference to yield a 98 gene *Lynx1*^−/−^ plasticity signature. FC, fold change. RP, rank product. pfp, percent false positive (i.e. false discovery rate).

Our efforts to understand the molecular machinery involved in the suppression of developmental plasticity focused on immediate changes in gene expression in V1 via acute inflammation (qPCR 4 h after a single intraperitoneal injection of LPS). We expected this time point to be particularly sensitive to disruption because the earliest experience-dependent changes occur within hours to a day of MD at the level of firing rate of parvalbumin interneurons (Aton et al., 2013; Kuhlman et al., 2013; Reh and Hensch, 2014) and protease (Mataga et al., 2002) and microglia activity (Sipe et al., 2016) as triggers for subsequent global ocular dominance plasticity, which takes a few days to be detected by single-unit recordings (Gordon and Stryker, 1996). Importantly, such trigger events occur only during the juvenile critical period when our assay was performed, but not in the adult period (Kuhlman et al., 2013). Thus, we reasoned that the baseline cortical expression signature at this early time point would be critical to gate global ocular dominance plasticity. In addition, peak acute inflammatory response, as measured by an increase in IL-1β in brain after peripheral LPS injection, is between 1 and 4 h (Layé et al., 1994; Eklind et al., 2006; Richwine et al., 2009), a time course well suited to disrupt these earliest experience-dependent plasticity events. While we speculate that LPS disrupted these early trigger events unique to juvenile cortex, more work needs to be performed to understand the molecular events underlying the functional changes seen within hours of MD and to dissect the impact of inflammation on these events. In addition to the early phase of plasticity, inflammation may also impact plasticity mechanisms during the later phases of MD because TNF-α is essential to nondeprived eye potentiation via a homeostatic mechanism at 5-6 d of MD (Kaneko et al., 2008). Ultimately, further work is necessary to tease out the interaction between inflammatory and plasticity mechanisms that contribute to the suppression of functional plasticity across multiple days of experience deprivation. Performing such work comparing acute versus chronic inflammatory models would provide fascinating insights into neuroimmune biology and would help to inform the important clinical question of the potential impact of acute and chronic inflammation on the neurodevelopmental trajectory in children.

While our experimental efforts focused on the impact of acute inflammation on plasticity, our list of diseases predicted to impact plasticity include diseases that accompany chronic inflammation (Table 3). Efforts to study human disease and animal models shed light on how acute versus chronic inflammation may affect plasticity. For example, components of plasticity and inflammation are dysregulated in epilepsy (Vezzani and Granata, 2005). Acute inflammation from low to high doses (LPS) decreases the threshold for the induction of seizure (Sayyah et al., 2003), and a single early life inflammatory insult increases susceptibility to seizure even into adulthood (Galic et al., 2008). Chronic overexpression of inflammation-related genes in rodents causes an increased or decreased susceptibility to seizure, depending on gene dose (Vezzani and Granata, 2005), and seizure itself appears to chronically induce inflammatory markers (De Simoni et al., 2000). This evidence indicates a potential bidirectional effect of epilepsy and inflammation, wherein acute and chronic inflammation may have immediate and long-term effects on epilepsy-related plasticity mechanisms. In addition to epilepsy, cortical lesions and hypoxia-ischemia induce a robust inflammatory response that can endure chronically (Bona et al., 1999; Schroeter et al., 2002) and disrupt ocular dominance plasticity weeks after the injury (Failor et al., 2010; Greifzu et al., 2011). Interestingly, anti-inflammatory (ibuprofen) treatment rescues MD-induced sensory learning (increased visual acuity of the nondeprived eye) in adult (P70–P110) after cortical injury via photothrombosis in the nearby primary somatosensory; however, ibuprofen did not restore ocular dominance plasticity (Greifzu et al., 2011). It is possible that the anti-inflammatory regimen or mechanism of action used was not sufficient to eliminate the inflammation and rescue ocular dominance plasticity, or it may reflect distinct mechanisms of plasticity and their modulation by inflammation in the juvenile cortex versus the adult. While it has been proposed that causes of plasticity disruption may be cortical deafferentiation in the case of cortical lesions or disruption of inhibitory interneurons in the case of hypoxia-ischemia, it is possible that inflammation downstream of injury disrupts plasticity and should be investigated further. In sum, there is evidence that chronic and acute inflammation go hand in hand with disrupted plasticity across a variety of brain disorders, on different time scales, and as a function of different underlying mechanisms. Going forward, work is necessary to understand the contribution of diverse inflammatory mechanisms in the disruption of various types of plasticity across a wide variety of neurological and neurodevelopmental conditions.

Our finding that inflammation suppresses developmental cortical plasticity suggests a potential public health concern related to neurodevelopmental trajectory. During the height of the critical period for visual plasticity (peak is 0.5–2 years in humans; Morishita and Hensch, 2008), children <5 years of age have the highest incidence of contracting LPS-carrying Gram-negative foodborne pathogens relative to other childhood or adult periods (Centers for Disease Control and Prevention, 2013). Other infections that induce inflammation also show an increased incidence during the peak of developmental plasticity in humans; >80% of children <3 years of age experience otitis media (ear infection; Marom et al., 2014) and children <5 years of age are hospitalized for influenza-related complications nearly an order of magnitude more often than children 5–17 years of age (incidence rate ratio = 8.1, 95% CI = 7.3–9.0; data are from Dawood et al., 2010). Our work suggests that this increased incidence of infection during postnatal periods of developmental plasticity (relative to older ages) may be an unrecognized mechanism by which inflammation alters the neurodevelopmental trajectory. Most directly, the suppression of visual cortex plasticity could disrupt the development of binocular matching (Wang et al., 2010), a process central to the development of normal vision that specifically depends on heightened plasticity during the critical period for visual development. In addition, higher-order cognitive processes could be disrupted, due to the hierarchical dependency of various critical periods of plasticity (Takesian and Hensch, 2013). In addition, given that mechanisms of plasticity identified in the visual critical period have translated to other brain regions and functions (Levelt and Hübener, 2012; Nabel and Morishita, 2013; Werker and Hensch, 2015), it is likely that inflammation could disrupt plasticity in other systems.

Our work is a natural extension into the postnatal epoch of the growing body of research indicating deleterious brain and behavioral outcomes due to prenatal inflammatory exposure (Choi et al., 2016; Steullet et al., 2016; Weber-Stadlbauer et al., 2017) and suggests that inflammation may have a more extensive impact on postnatal neurodevelopment and brain function than previously realized. In fact, childhood infections and inflammation are associated with subsequent diagnoses of autism, depression, and schizophrenia as well as declines in cognitive capacity (Dalman et al., 2008; Atladóttir et al., 2010; Khandaker et al., 2014; Benros et al., 2015). The elevated incidence rate of infections during childhood neurodevelopment (relative to older ages) and the association of childhood infection with subsequent neurodevelopmental disorder may indicate a partial explanation for the observed onset of psychiatric disorders in childhood and adolescence (Lee et al., 2014). Our work showing that inflammation disrupts developmental cortical plasticity suggests an unrecognized risk factor for neuropsychiatric disorders and provides a starting point to investigate the underlying pathophysiology.

We show here that an integrative bioinformatics approach is well suited to interrogate the interactions between disease processes and disruptions in developmental plasticity. To extend this approach further, molecular matching could be expanded to the >71,000 experiments publically available (as of 4 August 2016, there were 71,885 Gene Expression Omnibus “Series”), and Disease Leverage Analysis could be expanded to the universe of biologically defined gene sets (as of 4 August 2016, MSigDb alone contained 13,311 sets), facilitating more comprehensive interrogation of the disease space and generation of more specific hypotheses about disease processes that disrupt plasticity. Moreover, this approach is not limited to interrogating neurodevelopment but can be extended to other neurological signatures beyond plasticity. We expect it will be useful for identifying connections between disease processes and other brain phenotypes that can be appropriately represented by a transcriptional signature.

## Synthesis

Reviewing Editor: Margaret McCarthy, Univ of Maryland School of Medicine Decisions are customarily a result of the Reviewing Editor and the peer reviewers coming together and discussing their recommendations until a consensus is reached. When revisions are invited, a fact-based synthesis statement explaining their decision and outlining what is needed to prepare a revision will be listed below. The following reviewer(s) agreed to reveal their identity: Henner Koch.

Two reviewers appreciate the significance of the work but ask for several points of clarification as well as some moderation of the interpretation. There is no need for additional experiments but the authors should provide a comprehensive and detailed response to the reviewers comments and revise the manuscript accordingly.

Reviewer #1:

This manuscript applies bioinformatics to identify gene signatures correlated with the transcriptomic change occurring between P29 and >P90. They interpret their data posing that the change in plasticity occurring at the end of the critical period is the most important functional change associated with this transcriptomic change. They find that inflammation is the biological function in common between age-dependent regulation of transcription and many disease signatures. Moreover, inflammation associated pathways are also regulated in adult Lynx Ko mice that display juvenile OD plasticity. Finally they test the role of inflammation by inducing systemic inflammation with IP injection of LPS in P26 mice and finding an impaired plasticity.

The approach is original and the results would be interesting, however there are many parts especially concerning Fig 2-4 that are not clearly described in the text and make difficult to judge the data.

In particular in the results section describing fig. 3 it is stated that

"8 of 13 (61.5%) genes predicted to be anti-correlated to the plasticity signature showed a complete reversal in their differential expression pattern after LPS administration (all reversed genes p less than 5 x 10-4d, t-test of deltaCTs, n = 5 mice per group) (Figure 3)" It is unclear where is this prediction coming from. If it is from GSE3253 (only stated in fig legend) these data were obtained by LPS injection in aged mice. Lowel's lab showed that OD plasticity continues to decrease with age, and aged mice are particularly impervious to monocular deprivation. Is the anticorrelation present at the whole transcriptome level? Moreover, is the fact that 13 genes are reversed significant? Why validation (done in juvenile mice) excludes few of the 13 genes? For example, judging from fig. 4b RT-PCR analysis BDNF is downregulated by LPS, however fig 3b would predict upregulation.

In the analysis of the Lynx KO the authors claim that inflammation is again involved. However, it should be clarified whether the regulated genes are the same (or significantly enriched with) genes of the P29 vs adult comparison albeit with opposite regulation, or whether different genes (even if belonging to similar categories) are involved.

Considering the results from Lowel's lab that a cortical lesion impairs OD plasticity the LPS ephys data are not so unexpected although I accept that they are needed to logically close the argument.

"Mice that experienced opening of the sutured eye or that had poor recordings (less than 10 cells/mouse or less than 3 penetrations / mouse or lack of positive identification of monocular zone and secondary visual cortex) were excluded from further analysis". How did the author perform identification of monocular and secondary visual cortices?

Reviewer #2:

In this study the authors investigated the correlation between the transcriptome of a large number of diseases taken from public microarray data and the "plasticity signature" of the V1 area of the cortex (an area that is known to undergo a critical period with plasticity in juvenile mice). The plasticity signature was generated by comparing the transcriptome of juvenile and adult mice and also to adult Linx (-/-) mice (which have also a plasticity response in adulthood). They then used this plasticity signature to match it to the disease signatures of the public microarray data.In this first step the authors identified inflammation pathways to be related to a dysregulation of plasticity.

In a second step the authors used an experimental approach (LPS exposure) to verify their hypothesis concluded from the first step by artificially inducing an inflammatory response in mice and investigate, if similar signatures can be detected in the experimental model. Last the authors used an in vivo measurement of plasticity to show that the LPS exposure is suppressing the cortical plasticity response (in V1) in juvenile mice determined by a monocular deprivation test. The manuscript is well written and the overall findings of the study are interesting. The methods are appropriate and some important control experiments were performed. However, I have some concerns of the methods used and the interpretation of the data and think the caveats and limitations of the approach should be clearly stated in the discussion.

Concerns:

1) The samples taken to generate the set of disease signatures stem from public microarray data and are derived from different tissues and also species (Table 2). The authors cite and propose that the study by Dudley et al., 2009 showed, that this is a valid approach. However, even in this original study, the authors provide some hints for limitations of the approach: "We acknowledge several limitations to the approach taken by this study. Foremost, we acknowledge that experimental investigators will generally draw samples from tissues that are relevant to the disease condition under study. Therefore, we cannot assert that disease concordance would be maintained in samples drawn from tissues that would not commonly be chosen in the study of a disease". So I think also in the discussion of this study this should be clearly stated to give the reader a fair impression of the caveats of the approach.

2) The authors provide a nice experimental set to strengthen the hypothesis, that inflammation is sufficient to suppress plasticity in the model used. It would be interesting to know why the tissue samples were taken 4 hours after LPS injection and the in vivo measurements were done 3 days after LPS injection. Was it technically not possible to also take the tissue after the in vivo measurements? Maybe other signatures would be present at this time point? Also it might be that the plasticity response is still normal after 4 hours, since some plasticity changes occur only over longer time periods (i.e homeostatic plasticity, which was shown to be influenced by TNF-alpha and other inflammatory molecules.)

3) The identified diseases include acute and chronic diseases. Several studies have proposed chronic inflammation as a potential cause of dysregulation of plasticity. Posttraumatic epilepsy, for example, has been linked to a dysregulation of the plasticity due to inflammation of neuronal networks (De Simoni et al. 2000, Vezzani - 2005). It would be nice to discuss the differential effects of chronic vs. acute inflamation on plasticity.

4) Why did also diseases that have no clear neurological phenotype show a strong correlation in their transcriptomes found in this study. I think this also needs to be addressed in the discussion.
